# Process development and safety evaluation of ABCB5^+^ limbal stem cells as advanced-therapy medicinal product to treat limbal stem cell deficiency

**DOI:** 10.1186/s13287-021-02272-2

**Published:** 2021-03-19

**Authors:** Alexandra Norrick, Jasmina Esterlechner, Elke Niebergall-Roth, Ulf Dehio, Samar Sadeghi, Hannes M. Schröder, Seda Ballikaya, Nicole Stemler, Christoph Ganss, Kathrin Dieter, Ann-Kathrin Dachtler, Patrick Merz, Saadettin Sel, James Chodosh, Claus Cursiefen, Natasha Y. Frank, Gerd U. Auffarth, Bruce Ksander, Markus H. Frank, Mark A. Kluth

**Affiliations:** 1 TICEBA GmbH, Im Neuenheimer Feld 517, 69120 Heidelberg, Germany; 2 RHEACELL GmbH & Co. KG, Im Neuenheimer Feld 517, Heidelberg, 69120 Germany; 3 Department of Ophthalmology, Lions Eye Bank, University of Heidelberg, Heidelberg, Germany; 4 Department of Ophthalmology, University Hospital Heidelberg, Heidelberg, Germany; 5 Department of Ophthalmology, Massachusetts Eye & Ear, Harvard Medical School, Boston, MA USA; 6 Department of Ophthalmology, University Hospital Cologne, Cologne, Germany; 7 Center for Molecular Medicine Cologne (CMMC), University of Cologne, Cologne, Germany; 8 Department of Medicine, VA Boston Healthcare System, Boston, MA USA; 9 Division of Genetics, Brigham and Women’s Hospital, Harvard Medical School, Boston, MA USA; 10 Transplant Research Program, Boston Children’s Hospital, Harvard Medical School, Boston, MA USA; 11 Harvard Stem Cell Institute, Harvard University, Cambridge, MA USA; 12 The Schepens Eye Research Institute, Massachusetts Eye & Ear, Harvard Medical School, Boston, MA USA; 13 Department of Dermatology, Brigham and Women’s Hospital, Harvard Medical School, Boston, MA USA; 14 School of Medical and Health Sciences, Edith Cowan University, Perth, Western Australia Australia

**Keywords:** Advanced-therapy medicinal product, ABCB5, GMP manufacturing, Limbal stem cell deficiency, Limbal stem cells, p63, PAX6

## Abstract

**Background:**

While therapeutic success of the limbal tissue or cell transplantation to treat severe cases of limbal stem cell (LSC) deficiency (LSCD) strongly depends on the percentage of LSCs within the transplanted cells, prospective LSC enrichment has been hampered by the intranuclear localization of the previously reported LSC marker p63. The recent identification of the ATP-binding cassette transporter ABCB5 as a plasma membrane-spanning marker of LSCs that are capable of restoring the cornea and the development of an antibody directed against an extracellular loop of the ABCB5 molecule stimulated us to develop a novel treatment strategy based on the utilization of in vitro expanded allogeneic ABCB5^+^ LSCs derived from human cadaveric limbal tissue.

**Methods:**

We developed and validated a Good Manufacturing Practice- and European Pharmacopeia-conform production and quality-control process, by which ABCB5^+^ LSCs are derived from human corneal rims, expanded ex vivo, isolated as homogenous cell population, and manufactured as an advanced-therapy medicinal product (ATMP). This product was tested in a preclinical study program investigating the cells’ engraftment potential, biodistribution behavior, and safety.

**Results:**

ABCB5^+^ LSCs were reliably expanded and manufactured as an ATMP that contains comparably high percentages of cells expressing transcription factors critical for LSC stemness maintenance (p63) and corneal epithelial differentiation (PAX6). Preclinical studies confirmed local engraftment potential of the cells and gave no signals of toxicity and tumorgenicity. These findings were sufficient for the product to be approved by the German Paul Ehrlich Institute and the U.S. Food & Drug Administration to be tested in an international multicenter phase I/IIa clinical trial (NCT03549299) to evaluate the safety and therapeutic efficacy in patients with LSCD.

**Conclusion:**

Building upon these data in conjunction with the previously shown cornea-restoring capacity of human ABCB5^+^ LSCs in animal models of LSCD, we provide an advanced allogeneic LSC-based treatment strategy that shows promise for replenishment of the patient’s LSC pool, recreation of a functional barrier against invading conjunctival cells and restoration of a transparent, avascular cornea.

**Supplementary Information:**

The online version contains supplementary material available at 10.1186/s13287-021-02272-2.

## Introduction

The cornea maintains its transparency partly by continuously replacing aged or damaged epithelial cells. Physiological regular renewal of the corneal epithelium is guaranteed by stem cells residing in the limbal region. These limbal stem cells (LSCs) are crucial for corneal epithelial turnover and maintenance of a barrier between the clear, avascular cornea and the vascularized conjunctiva. Accordingly, LSC deficiency (LSCD), either due to congenital or, more frequently, acquired aplasia or depletion of LSCs by intrinsic or extrinsic insults of various etiologies [1], is characterized by compromised corneal epithelial regeneration and an impaired barrier function of the limbus. Under such circumstances, conjunctival epithelial cells can invade and successively replace corneal epithelial cells. As a result, corneal neovascularization, chronic inflammation, and stromal scarring can occur, which may contribute to discomfort, corneal opacification, vision loss, and even blindness [1–4]. According to a bulletin of the WHO, corneal disease is a major cause of blindness worldwide, second only to cataract [5].

Therapeutic options for LSCD depend on the etiology, severity of symptoms, extent (partial vs. total), and laterality (uni- vs. bilateral) of the disease [3, 4, 6, 7]. Treatment of mild and moderate cases aims at the control of symptoms. In these cases, recovery requires the presence of at least certain numbers of remaining LSCs that can restore the corneal epithelium. In severe LSCD, where no or insufficient amounts of LSCs are present, the LSC pool needs to be restored [4, 7]. Earlier procedures involved transplantation of limbal tissue, either from the patient’s healthy or less affected contralateral eye or, in cases of bilateral LSCD, from a living or deceased donor [8–10]. Newer, tissue-sparing techniques are based on transplantation of corneal grafts prepared from ex vivo cultured limbal cells (cultivated limbal epithelial transplantation) [11, 12] or transplantation of a limbal biopsy sample from the unaffected eye cut into small tissue pieces that are evenly distributed over an amniotic membrane scaffold attached to the affected eye’s corneal surface (simple limbal epithelial transplantation) [13–15]. These techniques have reduced the amount of donor tissue required and thus decreased the risk of harming the donor eye.

However, as LSCs comprise only a small population among heterogenous cell populations present in the limbus [16, 17] and transplantation success highly depends on the percentage of LSCs within the transplanted cells [18], a major challenge in the further development of transplantation techniques has remained: the prospective identification of LSCs, which would permit enrichment of the stem cell content of the transplant. Over decades, LSCs could only be identified retrospectively by indirect or functional characteristics including label retention; lack of expression of corneal differentiation markers such as cytokeratin (CK)3, CK12, and CK19; ability to generate holoclones; and corneal epithelial regeneration capacity after transplantation [19]. Furthermore, although the nuclear transcription factor tumor protein 63 (p63), specifically its N-terminally truncated alpha isoform ΔNp63α, had been shown to identify LSCs and was thus proposed as a direct LSC marker [20, 21], prospective cell sorting-based enrichment of limbal grafts for p63^+^ cells is not feasible, given its nuclear localization.

More recently, the membrane-bound ATP-binding cassette transporter, subfamily B, member 5 (ABCB5), originally described as a marker for dermal progenitor cells [22], was found to be expressed on label-retaining (slow-cycling), p63α^+^ CK12^−^ cells located in the basal limbal epithelium of mice and humans, respectively [23]. This discovery identified ABCB5 as the first molecular surface marker for prospective LSC enrichment by antibody-based cell sorting. Furthermore, *Abcb5* gene loss of function in *Abcb5*-knockout mice was associated with defective corneal differentiation and regeneration, indicating that ABCB5, beyond representing an LSC marker [24–27], is required for LSC function and corneal epithelial regeneration [23]. Purified CK12^−^ ABCB5^+^ LSCs could, in vitro, be induced to differentiate into CK12^+^ epithelial cells [28]. Moreover, in NSG mouse and New Zealand White rabbit models of surgically induced LSCD, grafts containing prospectively isolated human ABCB5^+^ limbal cells were able to restore corneal transparency and to provide a stratified, well-differentiated CK12^+^ corneal epithelium [23, 29].

Both the cell-surface localization of ABCB5 and the considerable cornea-restoring capacity of human ABCB5^+^ limbal cells suggest this cell population as a promising candidate for LSCD therapy. This stimulated us to develop a novel treatment strategy based on the utilization of allogeneic ABCB5^+^ LSCs that were derived from cadaveric ocular tissue, expanded in vitro and manufactured as an advanced-therapy medicinal product (ATMP). Here, we describe our Good Manufacturing Practice (GMP)-compliant manufacturing process, report on the preclinical safety testing of our LSC-based ATMP and present the treatment strategy that is currently being tested in a first-in-human clinical trial.

## Materials and methods

### Manufacturing of human ABCB5^+^ LSCs

#### Tissue procurement and processing

Cornea rims from human deceased donors were obtained from the Lions Eye Bank of the University Eye Clinic of Heidelberg, Germany, in cooperation with the German Society for Tissue Transplantation (DGFG), as leftover tissue from cornea transplantations. Tissues must fulfill the requirements laid down in the tissue regulation amending the German transplantation act (TPG-GewV). Donors who tested positive (serological and nucleic acid testing using pre- or postmortal blood) for HIV1/2, HBV, HCV, HTLV (if required), and/or *Treponema pallidum* were excluded. The manufacturing process took place in an EU-GMP grade A cabinet in a grade B clean room facility under laminar air flow (“A in B”) and followed GMP requirements. Tissue was disinfected, washed, freed from residual corneal and scleral tissue, dissected into fragments, and enzymatically dissociated via collagenase (Collagenase NB6, Nordmark, Uetersen, Germany) for 1.5–6 h at 37 °C in collagenase/PBS^Ca/Mg^/penicillin/streptomycin solution (containing 1 U/ml collagenase). Cells were centrifuged and cultured as unsegregated cell culture in a feeder cell-free stem cell-selecting medium (Dulbecco’s modified Eagle’s medium/Ham’s F-12 supplemented with fetal calf serum, l-glutamine, hydrocortisone, insulin, and recombinant human epidermal growth factor) on an uncoated 12-well plate in a cell culture incubator (5% CO_2_, 90% humidity, 37 °C).

#### Assessment of cell confluence and morphology

During cell expansion and isolation, cell confluence and morphology were assessed visually using phase-contrast microscopy by comprehensively trained lab assistants strictly employing the four eyes principle (i.e., cross-checked by the Head of Production).

#### Cell expansion and isolation

**Fig. 1:**
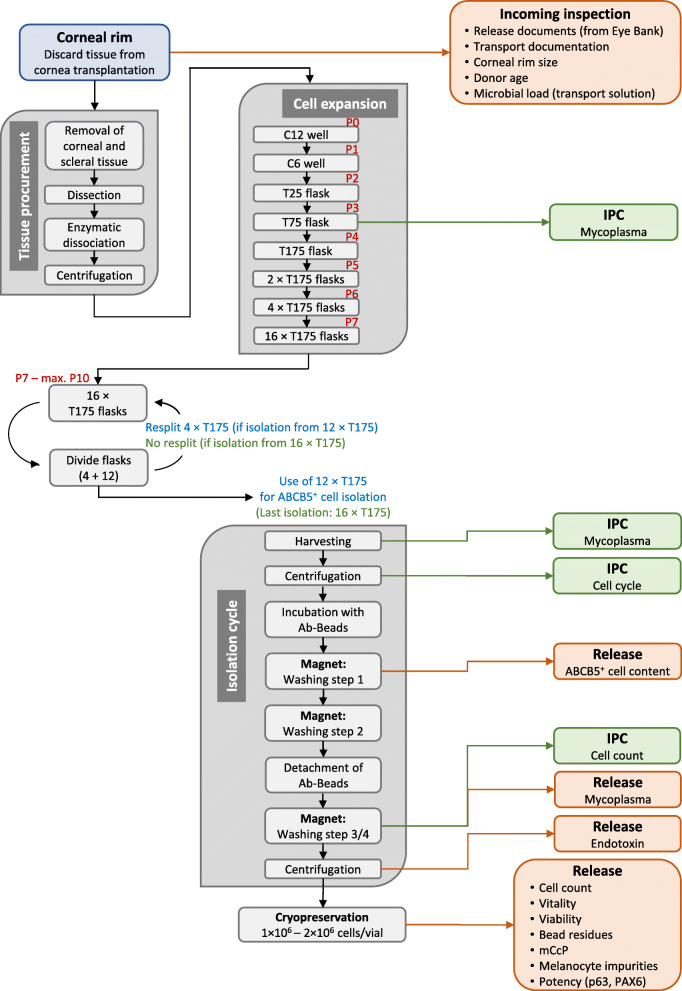
Flow chart summarizing the manufacturing process of human ABCB5^+^ limbal stem cells. In-process controls (IPCs) are colored in light green, release controls in orange. Due to lack of space, not all IPCs are shown (but given in Table 1). *BC* barcoded cryovial, *mCcP* microbiological control of cellular products

All expansion steps occurred in uncoated culture dishes using the medium described above. When ≥70% confluence was reached, cells were harvested using non-animal recombinant trypsin (TrypZean®, Sigma-Aldrich, Taufkirchen, Germany) and cultured on a 6-well plate. After subsequent expansion in a T25 flask and thereafter in T175 flasks for up to 10 passages in total (Fig. 1; representative morphological images of cultures at early and late passages see Figure S1 (Additional file 2)), ABCB5^+^ LSCs were isolated using magnetic polystyrene beads (micromer®-M, Micromod, Rostock, Germany) coated with a mouse anti-human monoclonal antibody directed against the 16-mer amino acid sequence 493–508 (RFGAYLIQAGRMTPEG) of the ABCB5 extracellular loop 3 [22] (bulk production: Maine Biotechnology Services, Portland, ME, USA; GMP purification: Bibitec, Bielefeld, Germany; virus depletion and safety study according to ICH Q5 [30]: Charles River, Erkrath, Germany). Briefly, cells were harvested by incubation with 0.02% EDTA in PBS, because trypsin harvest causes a transient loss of the epitope targeted by the antibody. After incubation of the cell suspension with the antibody-coated beads for 20–25 min, the ABCB5^+^ LSCS bound to the beads were magnetically separated from the unbound, ABCB5^−^ cells. Following enzymatic (TrypZean®) detachment of the beads from the cell surface, the isolated ABCB5^+^ LSCs were cryo-preserved in freeze medium containing 10% dimethyl sulfoxide as cryoprotectant (CryoStor® CS10, BioLife Solutions, Bothell, WA, USA) in polypropylene cryovials (1–2 × 10^6^ cells/cryovial) and stored in the vapor phase of liquid nitrogen at temperatures below − 130 °C. To maximize the yield of ABCB5^+^ LSCs from one corneal rim, up to four isolation cycles can be performed, provided that no changes in cell morphology or growth behavior (as assessed by phase-contrast microscopical inspection and confluence determination) occur (Fig. 1, Table 1). Several in-process controls (Table 1) and release controls (Table 2, Fig. 1) are performed to guarantee the quality of the isolated cells even after freezing/thawing (Table S1 (Additional file 1)).

**Table 1:** In-process controls during manufacturing of human ABCB5^+^ LSCs

Process step	Parameter	Criterion
Incoming good inspection^a^	Storage time of cornea before transplantation	≤ 34 days
	Storage time of the corneal rim in transport medium	≤ 4 days
	Size of the corneal rim	≥ ¾ of the rim
	Endothelial cell density of the cornea	≥ 2000 cells/mm^2^
	Donor serology (pre- or postmortal): HIV1/2, HBV, HCV, HTLV (if required), and *Treponema pallidum*	Negative
	Microbiological load of the transport medium	≤ 5 CFU/10 ml
Tissue dissociation	Dissociation efficiency after collagenase incubation	> 80%, 1.5–6 h
Cell expansion	First assessment of cell confluence	1–4 days
	Mycoplasma testing of medium supernatant (only on T75)	Not detectable (< 10 CFU/ml)
	Confluence before passaging	≥ 70%
	Cultivation time until passaging (12-well)	≤ 16 days
	Cultivation time until passaging (6-well, T25, T75, T175)	≤ 7 days
	Digestion efficacy after trypsin incubation	> 90%, 5–7 min
	Cell morphology	Undifferentiated cells, LSC morphology
Isolation of ABCB5^+^ LSCs	Confluence	70–95%
	Passage number	≤ 10
	Cultivation time since last passaging (last trypsin harvest)	≥ 3 days (to ensure presence of ABCB5 on cell surface)
	Efficacy of cell detachment from the culture vessel after incubation with Versene® (0.02% EDTA in PBS)	> 90%, 20–30 min
	Cell cycle profile (proportions of cells in G1, S, and G2/M phase	Determined and declared
	Cell count	n.a.
	Cell morphology	Undifferentiated cells, LSC morphology

*n.a.* not applicable

^a^Only corneal rims that fulfilled the requirements for transplantation (according to the tissue regulation amending the German tranplantation act (TPG-GewV) were used

**Table 2:** Specifications and results from GMP batch analysis (*n* = 13)

Parameter	Test method	Specification	Mean	Deviations from specification	
				Absolute	Percentage
ABCB5^+^ cell content	Flow cytometry	≥ 90%	96.4%	0	0%
Mycoplasma	Nucleic acid test-based assay (2.6.7 Ph. Eur.)	not detectable (< 10 CFU/ml)	< 10 CFU/ml	0	0%
Endotoxin level	LAL-Test (2.6.14 Ph. Eur.)	≤ 2 EU/ml	≤ 2 EU/ml	0	0%
Cell vitality	Flow cytometry (2.7.29 Ph. Eur.)	≥ 90%	97.5%	1	7.7%
Cell viability	Flow cytometry (2.7.29 Ph. Eur.)	≥ 90%	99.2%	0	0%
Bead residues	Flow cytometry	≤ 0.5%	0.04%	0	0%
Microbiological control (*n* = 12)^a)^	BacT/ALERT® System (adapted to 2.6.27 Ph. Eur.)	no growth	n.a.	0	0%
p63^+^ cell content	Immunofluorescence	≥ 20%	76,7%	0	0%
PAX6^+^ cell content (*n* = 8)^b)^	Immunofluorescence	≥ 50%	71.3%	0	0%

*Ph. Eur.* European Pharmacopeia, *LAL* Limulus amebocyte lysate, *n.a.* not applicable

^a^One batch could not be evaluated due to sample size error and was therefore not released

^b^Five batches could not be evaluated due to staining problems and were therefore not released

### Characterization of human ABCB5^+^ LSCs

#### Proliferation assay

Cells were labeled with carboxyfluorescein diacetate succinimidyl ester (CFSE) using the Invitrogen™ CellTrace™ CFSE proliferation kit (Thermo Fisher, Langenselbold, Germany) according to the manufacturer’s instructions, and CFSE fluorescence was measured over time by flow cytometry.

#### Immunofluorescence staining

Cryosections of the human limbal and corneal tissue (4 μm) were fixed in 4% paraformaldehyde (PFA) and stained for ΔNp63, p63α, or CD1a (for antibodies see Table S2 (Additional file 1); incubation time 40–50 min for primary and 30–35 min for secondary antibodies). Prior to p63 staining, sections were permeabilized using 1% Triton™ X-100 (Sigma-Aldrich) in phosphate-buffered saline (PBS) (10 min). Nuclei were counterstained with 4′,6-diamidino-2-phenylindole (DAPI) (10–12 min) and stains microscopically evaluated.

Cells were seeded or centrifuged (Cytospin™; Thermo Fisher) onto microscope slides, fixed (4% PFA) and stained for ΔNp63, CK3/CK12, CK19, paired box protein 6 (PAX6), vimentin, connexin 43, melanoma antigen recognized by T cells (MART-1) and CD1a, respectively (for antibodies see Table S2 (Additional file 1); incubation time 40–50 min for primary and 30–35 min for secondary antibodies). Prior to staining of intracellular proteins, cells were permeabilized with 1% Triton™ X-100/PBS (10 min). Nuclei were counterstained with DAPI (10–12 min). For positive control, human corneal epithelial cells (Life Technologies, Darmstadt, Germany; for ΔNp63, CK3/CK12, PAX6), human skin-derived mesenchymal stem cells (TICEBA [31]; for vimentin, connexin 43) and human skin malignant melanoma cells (SK-MEL-28, ATCC® HTB-72™, LGC Standards, Wesel, Germany; for MART-1) were used. Stains were evaluated microscopically (Leica DMi8 microscope, Leica Microsystems, Wetzlar, Germany; or Floid™ cell imaging station, Life Technologies, Darmstadt, Germany).

#### Measurement of VEGF secretion

ABCB5^+^ LSCs were cultured for 48 h under hypoxic conditions (1% O_2_, 4% CO_2_, 95% N_2_) or in fibrin gel (Tisseel®; Baxter, Unterschleißheim, Germany). Vascular endothelial growth factor (VEGF) concentration in cell culture supernatant was measured using the Invitrogen™ VEGF Human ELISA Kit (Thermo Fisher) according to the manufacturer’s instructions.

### Batch analyses

Batch analyses followed validated GMP-compliant procedures according (where applicable) to the requirements of the European Pharmacopeia. For an overview and specifications see Fig. 1 and Table 2, respectively.

#### Determination of ABCB5^+^ cell content

After the isolation of the ABCB5^+^ cells, but before the enzymatic detachment of the microbeads (which leads to transient loss of ABCB5 from the cell surface), ABCB5^+^ cell content was determined following incubation (20–30 min) with an Alexa Fluor® 647-coupled donkey anti-mouse secondary antibody (Invitrogen/Thermo Fisher, Cat. # A-31571) targeting the anti-ABCB5 antibody used for cell isolation. To discriminate between ABCB5^+^ cells and free bead-antibody complexes, calcein acetoxymethylester was added to the cell suspension before incubation. Calcein and Alexa Fluor® 647 fluorescence was measured using a BD Accuri™ C6 Flow Cytometer (Becton Dickinson, Heidelberg, Germany). By gating only events with high calcein fluorescence (indicative of viable cells), unbound bead-antibody complexes were excluded from the ABCB5^+^ cell content calculation (for gating strategy see Figure S2 (Additional file 2)).

#### Mycoplasma testing

Cell suspension samples were spiked with internal control DNA, and genomic DNA was isolated using the Microsart® AMP Extraction Kit (Minerva Biolabs, Berlin, Germany). Isolated DNA was subjected to TaqMan® qPCR using the Microsart® ATMP Mycoplasma qPCR Kit (Minerva Biolabs), which includes positive and negative controls and 10CFU™ Sensitivity Standards for *Mycoplasma (M.) orale*, *M. fermentans* and *M. pneumoniae*.

#### Endotoxin testing

After the ABCB5^+^ cell isolation, microbead detachment and washing/centrifugation of the cell suspension, supernatant was diluted 1:10 with Endosafe® (Charles River, Charleston, SC, USA) Limulus Amebocyte Lysate Reagent Water and transferred into an Endosafe® PTS™ cartridge, which was loaded into an Endosafe® PTS™ reader. The endotoxin level was calculated based on the change in optical density analyzed against an internal standard curve.

#### Determination of cell count and vitality

To stain dead cells, propidium iodide solution (1 mg/ml) was added to cell suspension samples in PBS containing 2 mM ethylenediaminetetraacetic acid (EDTA) and 1% human serum albumin and incubated for 2 min. Fluorescence was measured using a BD Accuri™ C6 flow cytometer, and cell count and vitality were calculated.

#### Determination of cell viability and bead residues

Viability and microbead residues, which might result from insufficient bead detachment or cell washing, were analyzed in parallel. Cell suspension samples were incubated for 20–30 min at 37 °C with calcein acetoxymethylester to stain metabolically active cells. Calcein fluorescence was measured by flow cytometry (BD Accuri™ C6) and viability was calculated. For detection of residual microbeads, a cell-free solution of ABCB5 antibody-conjugated beads was used to define a gate in the FSC/SSC dot plot. To exclude false-positive signals from viable cells, only calcein-negative events in that gate were counted for calculation of bead residues.

#### Microbiological examination

Microbiological examination was performed by a certified academic contract laboratory. Cell suspension samples were diluted with NaCl-peptone buffer solution, inoculated in BacT/ALERT® (bioMérieux, Nürtingen, Germany) BPN (anaerobic) and BPA (aerobic) culture bottles, and incubated in the BacT/Alert 3D60 automated microbial contamination detection system. Positive samples were seeded onto solid culture medium immediately after detection. After 7 days of incubation, all negative samples were seeded onto solid culture medium.

#### Determination of p63^+^ and PAX6^+^ cell content (potency assays)

Cells (5 × 10^3^) were either centrifuged (Cytospin™) onto microscope slides or cultured for several hours on coverslips, fixed (4% PFA), permeabilized (1% Triton™ X-100/PBS, 10 min), and stained for ΔNp63 or PAX6, using antibodies as specified in Table S2 (Additional file 1) (incubation times 40–50 min for primary and 30–35 min for secondary antibodies). Nuclei were counterstained with DAPI (10–12 min) and slides mounted with Vectashield® (Vector Laboratories, Peterborough, UK). Images were captured using a Leica DMi8 microscope or a Floid™ cell imaging station and percentages of ΔNp63^+^ and PAX6^+^ cells among DAPI-positive cells calculated. For positive and negative control, cryosections of human corneal rim samples were stained accordingly. Assays were considered valid if the limbal tissue exhibited ≥20 DAPI^+^ cells per field of view that were also p63^+^ and PAX^+^, respectively, while the scleral tissue exhibited ≤5% p63^+^ and PAX6^+^ cells, respectively, among DAPI^+^ cells.

#### Control for melanocyte contamination

Cells (5 × 10^3^) were centrifuged (Cytospin™) onto microscope slides, fixed (4% PFA), permeabilized (1% Triton™ X-100/PBS, 10 min), and stained for MART-1 (for antibodies see Table S2 (Additional file 1); incubation times 40–50 min for primary and 30–35 min for secondary antibody). Nuclei were counterstained with DAPI (10–12 min) and slides mounted with Vectashield®. Images were captured using a Leica DMi8 microscope or a Floid™ cell imaging station. SK-MEL-28 cells (5 × 10^3^), stained accordingly, served as positive control.

### Animal studies

#### Animals

NSG (NOD.*Cg-Prkdc*^*scid*^*Il2rg*^*tm1Wjl*^/SzJ) mice were supplied by Charles River (Saint-Germain-Nuelles, France; local biodistribution and toxicity study) or Jackson (Bar Harbor, ME, USA; systemic biodistribution and toxicity/tumorigenicity studies). At initiation of treatment, animals were 6–8 weeks old. Mice were housed individually under special hygienic conditions with 12-h/12-h light-dark cycle, fed ad libitum with a rodent complete diet and had free access to drinking water. All animal experiments were performed by specialized contract research organizations in France (local biodistribution and toxicity study), meeting the animal protection requirements defined in the European [32] and French animal welfare legislations, and the USA (systemic biodistribution and toxicity/tumorigenicity studies), meeting all relevant animal welfare regulation and strictly adhering to the animal welfare standards defined by the U.S. Department of Agriculture (USDA) [33], National Research Council [34], Office for Laboratory Animal Welfare (OLAW) [35], ISO 10993-2 [36], and Association for the Assessment and Accreditation of Laboratory Animal Care (AAALAC). All experimentation procedures had been approved by the competent national authorities and institutional boards, as applicable. For an overview over all animal studies see Table 3.

**Table 3:** Overview over the preclinical in-vivo safety studies of ABCB5^+^ LSCs

Mouse strain	Number of animals	Model	Cell dose	Route and time of application	Observation period
**Local biodistribution study**					
NSG	10 (5 males, 5 females)	Corneal and limbal debridement [23]	5000 ABCB5+ LSCs in fibrin gel	Topical, day 0	8 weeks
**Systemic biodistribution study**					
NSG	30 (15 males, 15 females)	healthy	0.5 × 10^6^ ABCB5^+^ LSCs in HRG solution	Subconjunctival, day 0	1 week (*n* = 10)
					12 weeks (*n* = 10)
					20 weeks (*n* = 10)
**Single-dose local toxicity study**					
NSG	20 (10 males, 10 females)	Corneal and limbal debridement [23]	5000 ABCB5+ LSCs in fibrin gel (*n* = 10)	Topical, day 0	8 weeks
			Carrier (fibrin gel) only (*n* = 10)		
**Systemic repeated-dose toxicity and tumorigenicity study**					
NSG	50 (25 males, 25 females)	healthy	0.5 × 10^6^ ABCB5^+^ LSCs in HRG solution (*n* = 20)	Subconjunctival, days 0, 14, 28	20 weeks
			Vehicle (HRG solution) only (*n* = 20)	Subconjunctival, days 0, 14, 28	
			1.25 × 10^4^ HeLa cells in BSS solution (*n* = 10)	Subconjunctival, day 0	

*LSC* limbal stem cell, *LSCD* limbal stem cell deficiency, *HRG* Ringer’s lactate solution human serum albumin and glucose, *BSS* balanced salt solution

#### Corneal and limbal debridement

Animals were anesthetized by intraperitoneal injection of ketamine (Imalgene® 500, Merial, Lyon, France; 50 μl/male, 40 μl/female) and xylazine (Rompun® 2%, Bayer, Lyon, France; 50 μl/male, 40 μl/female). For analgesia, mice received meloxicam (Mobic® 15 mg/1.5 ml, Boehringer Ingelheim, Paris, France; 100 μl/animal subcutaneously) and tetracaine 1% (TVM, Lempdes, France; 1 drop into the right eye). The limbal and corneal epithelium of the right eye was removed with an Algerbrush II rust ring remover, working in a circular motion starting at the central cornea. After debridement, the eye was rinsed with 35% ethanol followed immediately by normal saline. A neomycin- and polymyxin B-containing eye ointment (Cebemyxine®, Bausch & Lomb, Montpellier, France) was applied for local antibiotherapy. For post-surgery pain relief, mice received subcutaneous injections of buprenorphine (Buprecare® 0.3 mg/ml, Axience, Pantin, France; 100 μl twice daily for 2 days) and meloxicam (Mobic® 15 mg/1.5 ml; 100 μl/animal at 48 h post-debridement).

#### LSC transplantation

Transplantation of ABCB5^+^ LSCs was carried out using Tisseel® fibrin sealant kit. Both, the fibrinogen and thrombin components were thawed and 500 μl of each solution diluted by adding 250 μl normal saline. ABCB5^+^ LSCs were thawed, washed, and suspended in HRG solution (Ringer’s lactate solution containing 2.5% human serum albumin and 0.4% glucose) at a concentration of 2 × 10^5^ cells/ml. Cell suspension (500 μl, containing 1 × 10^5^ ABCB5^+^ LSCs) was centrifuged, the supernatant removed, and the cells suspended in 20 μl of the fibrinogen/saline solution, yielding a concentration of 5000 cells/μl.

Matrix graft application was performed 2 days after corneal debridement under anesthesia and analgesia as described above. The right eye was proptosed and 1.5 μl of the thrombin/saline solution applied to the central cornea using a pipette and gently spread to the limbus using the pipette tip. Then, 1 μl fibrinogen/saline solution (containing 5000 ABCB5^+^ LSCs; control animals: without cells) was dropped onto the central cornea. After polymerization, tarsorrhaphy (8–0 suture) was performed. Post-transplantation treatment included local anti-inflammatory (dexamethasone; Maxidex® eye drops, Alcon, Rueil-Malmaison, France) and antibiotic (neomycin/polymyxin B; Cebemyxine®) care immediately after transplantation and on the day thereafter and pain relief (meloxicam; Mobic® 15 mg/1.5 ml; 100 μl/animal 24 h post-transplantation). Tarsorrhaphy suture was removed at 6–7 days after graft application under anesthesia as described above.

#### Subconjunctival cell injections

ABCB5^+^ LSCs were thawed, washed, and suspended in HRG solution at a concentration of 2 × 10^7^ cells/ml. HeLa cells (ATCC CCL-2) were cultured in Eagle’s minimum essential medium containing 10% fetal bovine serum, 2 mM glutamine, 1 mM sodium pyruvate, 100 U/ml penicillin, and 100 μg/ml streptomycin and passaged at least twice. On the day of application, HeLa cells were harvested using trypsin/ETDA solution, pelleted by centrifugation, and then rinsed in balanced salt solution (BSS) before being resuspended in BSS at a concentration of 0.5 × 10^6^ cells/ml. The injection volume (25 μl) was drawn up into a sterile 25-μl Hamilton syringe connected to a sterile 26G needle, and the syringe gently inverted several times to ensure homogenous suspension.

For injection, mice were anesthetized (1–3% isoflurane) and received topical anesthesia of the right eyeball surface by one drop of proparacaine hydrochloride ophthalmic solution. Cell suspension was injected subconjunctivally at the inferior conjunctival sac at a rate of 10 μl/s. After injection, erythromycin 0.5% ophthalmic ointment or BSS was applied to the surface of both corneas to maintain moisture. For pain relief, animals received 0.01–0.05 mg/kg buprenorphine hydrochloride (Buprenex®, Reckitt Benckiser) before and after subconjunctival injection.

#### Ophthalmic examinations

Ophthalmic examinations were carried out under general anesthesia (1–3% isoflurane). Examinations included assessment of the corneal surface using fluorescein staining (local toxicity study only), assessment of the cornea, conjunctiva, iris, anterior chamber and lens using slit lamp examination, and examination of adnexa, optic media, and fundus using indirect ophthalmoscopy. To facilitate the fundus examination, the pupils were dilated by instillation of tropicamide 0.5% or 1%.

#### Histopathology

In the local toxicity study, histopathological examinations were performed on the eyes with the eyelids attached, optic nerves, Haderian glands with intra-orbital lacrimal glands, extra-orbital lacrimal glands, nasal cavity, and brain. Tissues were fixed and preserved in modified Davidson’s fixative (nasal cavity and brain: 10% neutral-buffered formalin) for 48–72 h and then transferred to ethanol 70% or embedded or decalcified. The nasal cavity was sectioned at two levels as described by Uraih and Maronpot [37], including the nasolacrimal ducts. Each eye was sectioned at 12 levels. In the systemic toxicity/tumorgenicity study, all organs and tissues designated for histopathological assessment (see “Results” section) were harvested, fixed in 10% neutral-buffered formalin, and embedded in paraffin.

Sections were stained with hematoxylin/eosin. Additionally, sections from the debrided eyes were stained with Alcian Blue/Periodic acid–Schiff. Stained sections were examined by light microscopy. The observations were semi-quantitatively quantified as grade 1 (minimal/very few/very small), 2 (slight/few/small), 3 (moderate/moderate number/moderate size), 4 (marked/many/large), and 5 (massive/severe/very many/very large).

#### Quantitative polymerase chain reaction (qPCR)

Human ABCB5^+^ LSCs in mouse tissues were detected and quantified using a TaqMan®-based qPCR assay that had been validated according to the bioanalytical method validation guideline of the European Medicines Agency [38]. The following tissues were collected: local biodistribution study: anterior eye segment (cornea and lens), posterior eye segment (retina, sclera, and optic nerve), surrounding eye tissues (eyelids, lacrimal canals, and extra-orbital lacrimal glands), and muzzle with nasal cavities and naso-lacrimal duct; systemic biodistribution study: the blood, the femur bone with the bone marrow, brain, kidneys, liver, lungs, lymph nodes near to the injection site, ovaries or testes, skeletal muscle, skin/subcutis, spleen, thymus, thyroid/parathyroid gland, muzzle with nasal cavities and nasolacrimal duct, treated eye, untreated eye, surrounding ocular tissue (eyelid, lacrimal canals, extraorbital glands) of the treated eye, and surrounding ocular tissue of the untreated eye.

Assays were performed by a specialized contract research service provider. Tissues were homogenized using the Precellys® Evolution homogenizer (Bertin Technologies, Frankfurt, Germany) at 6800 rpm for at least two cycles (20 s each) at room temperature with at least 30 s pause between cycles. DNA was extracted using NucleoSpin® 96 Tissue kit (Macherey-Nagel, Düren, Germany). For primers and probes used for detection of human and mouse (to control for quality of the isolated DNA) DNA sequences see Table S3 (Additional file 1). Amplifications were performed on an Applied Biosystems™ ViiA™ 7 Dx Real-Time PCR instrument.

#### Statistics

Data acquisition and analysis were carried out using the Provantis® Version 9 preclinical software suite (Instem, Conshohocken, PA, USA). In the local biodistribution and toxicity studies, data transformation (none, log, or rank) was based on the kurtosis of the data, and the results of a Bartlett’s test for variance homogeneity and similarity of group sizes. Non- or log-transformed data were analyzed by parametric, rank-transformed data by non-parametric methods. Homogeneity of means was assessed by analysis of variance (ANOVA). Groups were compared using the two-sample *t* test (for parametric data) or the Mann-Whitney *U* test (for non-parametric data). In the systemic biodistribution and toxicity/tumorgenicity studies, quantitative, continuous data were analyzed using one-way ANOVA. Differences between groups were considered statistically significant if *p* ≤ 0.05.

### Clinical trial

#### Study design

A non-controlled, international, multicenter phase I/IIa clinical trial (ClinicalTrials.gov NCT03549299) was designed to evaluate the safety and efficacy of ascending doses of allogeneic ABCB5^+^ LSCs for the treatment of LSCD. Main inclusion and exclusion criteria are given in Table S4 (Additional file 1). It is planned to treat 16 patients at several sites in Germany and the USA. The trial was approved by the relevant independent ethics committees/institutional review boards and by the Paul Ehrlich Institute and the U.S. Food and Drug Administration, respectively, as the competent national regulatory authorities.

#### Interventions

Following surgical dissection of conjunctival pannus tissue from the corneal surface, 300 μl HRG solution containing 7.5 × 10^4^, 3 × 10^5^, 8 × 10^5^, or 1.2 × 10^6^ allogeneic ABCB5^+^ LSCs is evenly applied onto the entire corneal and limbal area. Cells are immediately fixed by means of a fibrin gel (Tisseel®), which has been successfully used as a glue fixative in limbal stem cell transplantation [12, 39–41]. After polymerization of the fibrin gel, the eye is covered with a bandage contact lens and a symblepharon shell to hold the graft in place and then bandaged using a perforated plastic shield.

For pre- and post-operative local anti-angiogenic therapy, patients receive subconjunctival injections of 75 μl (25 mg/ml) anti-VEGF antibody bevacizumab (Avastin®, Roche) at each quadrant 1 week before, directly after, and 2, 4 and 6 weeks following LSC transplantation. Concomitant immunosuppressive medication includes topical and systemic corticosteroids and long-term ciclosporin (months 1–6: orally, Sandimmun® Optoral/Sandimmune® soft gelatin capsules, Novartis; months 7–12: topically, Ikervis® eye drops, Santen/Restasis® ophthalmic emulsion, Allergan).

#### Outcome measures

Each patient will be followed up for 24 months. Primary efficacy endpoint of the clinical trial is defined as the response rate at 12 months after LSC transplantation, with response defined as no or mild corneal neovascularization and no or mild epithelial defects. Secondary efficacy measures include the response rate at 3 months after LSC transplantation and neovascularization, epithelial defects, ocular symptoms (pain, photophobia, burning), ocular inflammation, corneal opacity, visual acuity, and quality of life (as per visual function questionnaire-25) measured at various pre-defined time points throughout 12 months after LSC transplantation. Primary safety measures are adverse events throughout 24 months after LSC transplantation. Secondary safety measures include physical examinations, vital signs, and tonometry.

## Results

### Process validation

#### Tissue processing

Dissociation of corneal rim fragments via collagenase digestion was superior over dispase digestion or no enzymatic treatment (not shown). Collagenase digestion (1.5–6 h at 37 °C in collagenase/PBS^Ca/Mg^/penicillin/streptomycin solution containing 1 U/ml collagenase) resulted in cell yields between 7.5 × 10^4^ and 1.06 × 10^6^ cells (Figure S3a, left (Additional file 2)). Mean cell adhesion rate after collagenase digestion was about 30% (Figure S3a, right (Additional file 2)). Both cell count and adhesion rate after collagenase digestion were not dependent on donor age and tissue storage time (i.e., time between death of the donor and digestion of the corneal rim), respectively (Figure S3b (Additional file 2)).

#### Percentage of ABCB5^+^ cells throughout cell expansion process

Percentage of ABCB5^high^ cells, as determined by flow cytometry, within cultured unsegregated cells of different passages from 9 donors (*n* = 19 samples in total) was about 17.5% on average (Figure S3c (Additional file 2)). After passage 10, a decrease in the percentage of ABCB5^+^ (ABCB5^med/high^) cells became significant obvious (*n* = 1–10 donors; Figure S3d (Additional file 2)). As a consequence, the maximum passage number was set to 10.

### Characterization of cultured ABCB5^+^ LSCs

#### Growth behavior of cultured ABCB5^+^ LSCs

LSCs from four donors were labeled with CFSE and fluorescence measured during 9–11 days of culture. In parallel, cell confluence was visually evaluated under a phase contrast microscope, and cell count was determined. As shown in Figure S4 (Additional file 2), ABCB5^+^ LSCs slowed down proliferation when having reached confluence. No overgrowing potential could be detected within this period.

#### Marker expression profile of unsegregated and ABCB5^+^ limbal cells

Immunofluorescence evaluation of unsegregated cultured limbal cells (*n* = 6 cultures > 8 passages) revealed homogenous expression of PAX6, heterogenous expression of ΔNp63, slight expression of vimentin, connexin 43, negligible expression of CK19, and total absence of CK3/12 (Figure S5 (Additional file 2)). For validation of the ΔNp63 antibody, sections of human limbus and cornea were stained, showing positivity only in a small population of cells in the basal layer of the limbus, but not in the cornea (Figure S6 (Additional file 2)).

**Fig. 2:**
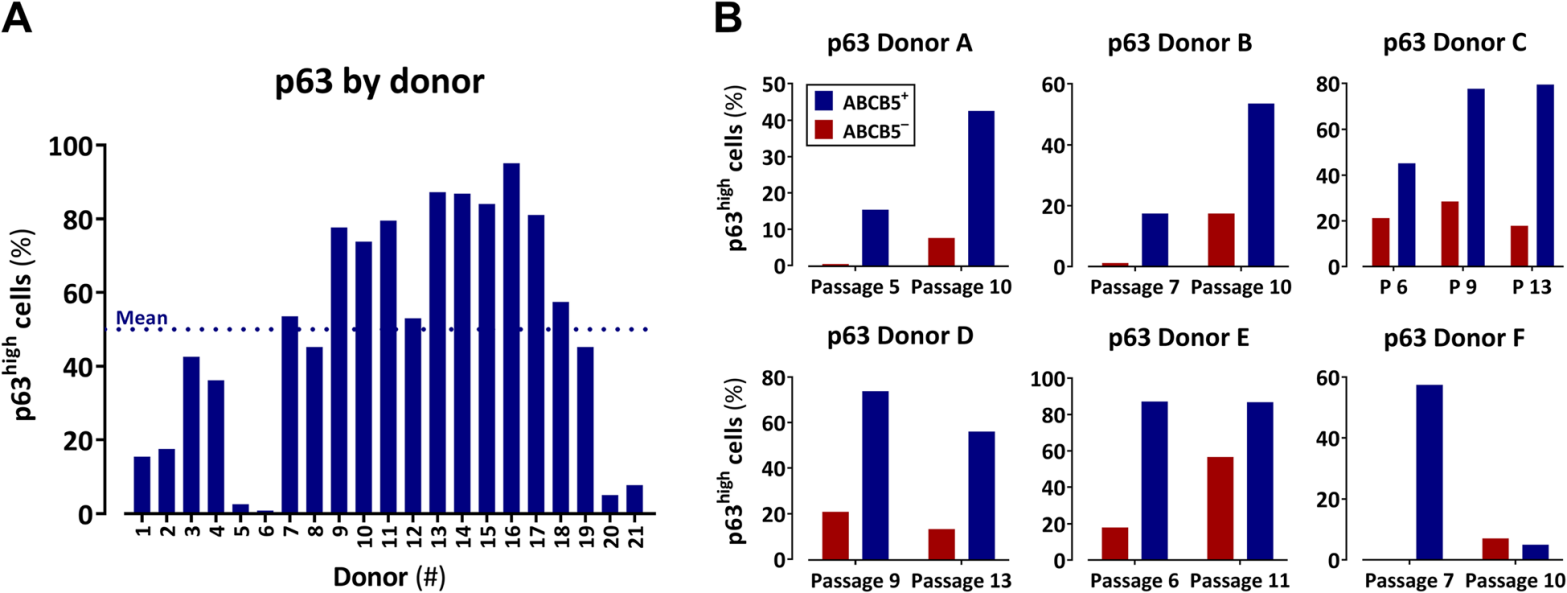
Correlation between ABCB5 and p63 expression in culture-expanded limbal stem cells. **a** Flow cytometric (rabbit anti-human p63, clone H137, AB-653763; Santa Cruz Biotechnology, Heidelberg, Germany, Cat. No. sc-8343) measurement of the percentage of p63^high^ cells within magnetic bead-isolated ABCB5^+^ cells from different donors. **b** Percentage of p63^high^ cells within ABCB5^+^ (blue) as compared to ABCB5^−^ (red) cells from various passages

**Fig. 3:**
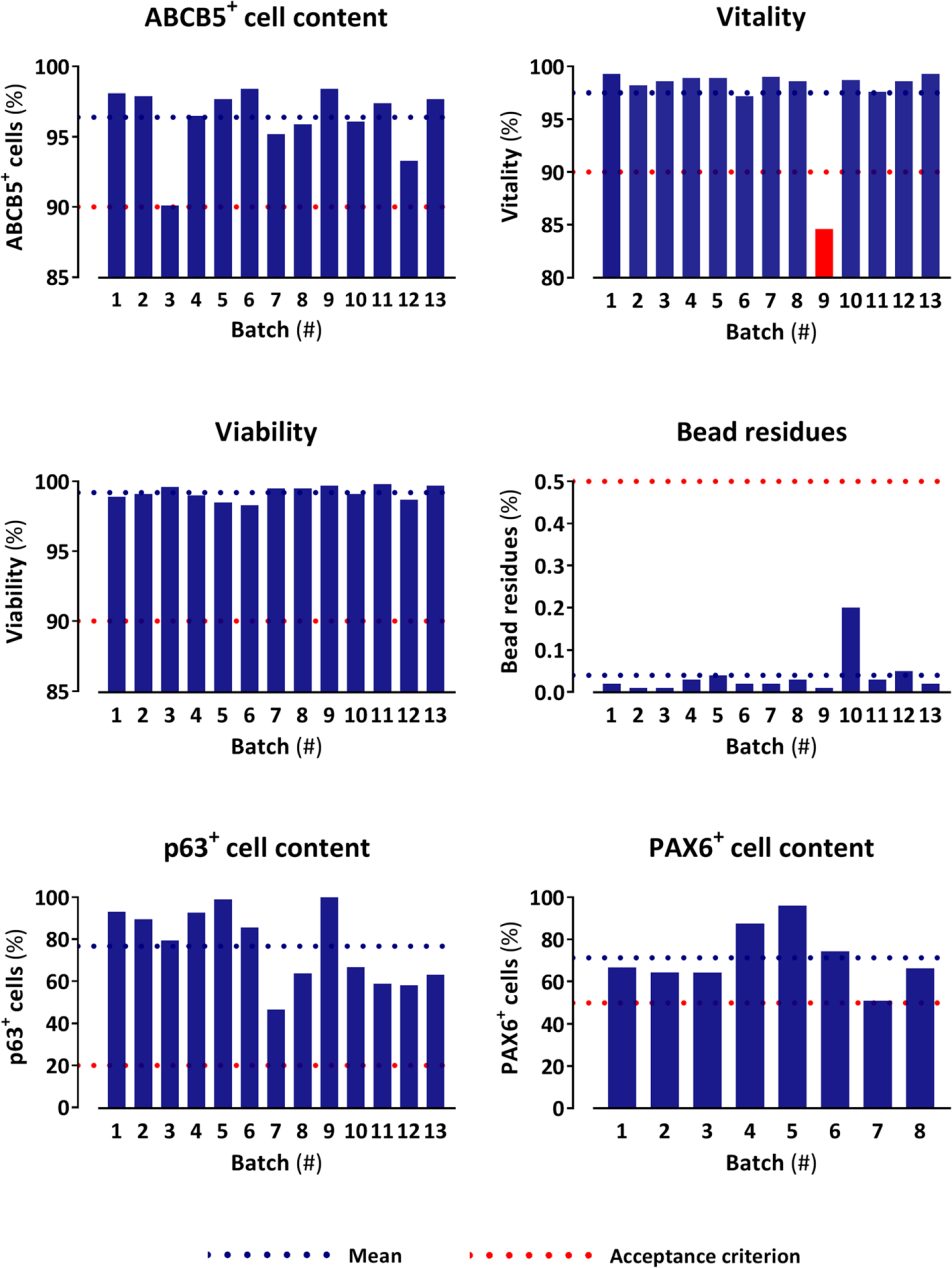
Release tests of 13 GMP-conform produced limbal stem cell batches. A batch that failed the specification for batch release is colored in red. Please note that batches 9–13 could not be evaluated for PAX6^+^ cell content due to staining problems and were therefore not released

Since p63 expression was linked to clinical transplant success [18], we investigated the correlation between expression of ABCB5 and p63 of LSCs from different donors and different passages. Flow cytometric determination of p63^high^ cells among bead-isolated ABCB5^+^ LSCs from 21 donors revealed a mean p63^high^ cell content of 50% (Fig. 2a). Within donors, the percentage of p63^high^ cells was highly enriched in the ABCB5^+^ population as compared to the ABCB5^−^ population and did, in general, not decrease with increasing passage number (Fig. 2b). Quality control data from GMP-compliantly manufactured cell batches, where the p63^+^ cell content was determined by immunofluorescence staining (i.e., without discriminating between high and low expressing cells), show a mean p63^+^ cell content of 77% (Table 2, Fig. 3).

To investigate whether the bead-isolated ABCB5^+^ LSCs express the p63 isoform ΔNp63α recognized as LSC marker [21], for which, however, no specific antibody is commercially available, double-staining for N-terminally truncated p63 (ΔNp63) and p63 alpha isoforms (p63α) was performed. In corneal tissue sections, a cell population located in the limbal region stained positive with both antibodies (Figure S7a-d (Additional file 2)). Among bead-isolated ABCB5^+^ LSCs, 66%, 64%, and 55% of nuclei stained positive for ΔNp63, p63α, and both, respectively (Figure S7e-h (Additional file 2). Thus, about 83% of the cells that stained positive for ΔNp63 were also positive for p63α.

#### Control for residual melanocytes cells and Langerhans cells

To detect potential impurities by residual melanocytes/melanoma cells and Langerhans cells, the final drug product (ABCB5^+^ LSCs) was evaluated by immunofluorescence staining for MART-1 (*n* = 4 batches) and CD1a (*n* = 3 batches), respectively. Whereas SK-MEL cells as positive control stained highly MART-1-positive, MART-1 positivity could not be detected in the final drug product. Likewise, no CD1a^+^ cells could be detected, neither in cryosections of the corneal rim as the starting material for LSC production nor in the final drug product. Flow cytometric analysis of 4 batches of the final drug product for CD1a^+^ cells revealed a mean CD1a^+^ cell content of 0.2 ± 0.3% (not shown).

### Batch quality control

Batch analyses including ABCB5^+^ cell content, mycoplasma testing, endotoxin level, cell vitality and viability, bead residues, microbiological examination, and PAX6^+^ and p63^+^ cell content were performed on 13 LSC batches (except for microbiological examination, *n* = 12, and PAX6^+^ cell content, *n* = 8). Except for one batch, which failed the specification for vitality, all batches fulfilled the specifications defined for batch release (Table 2 and Fig. 3).

### Behavior of ABCB5^+^ LSCs in fibrin gel

**Fig. 4:**
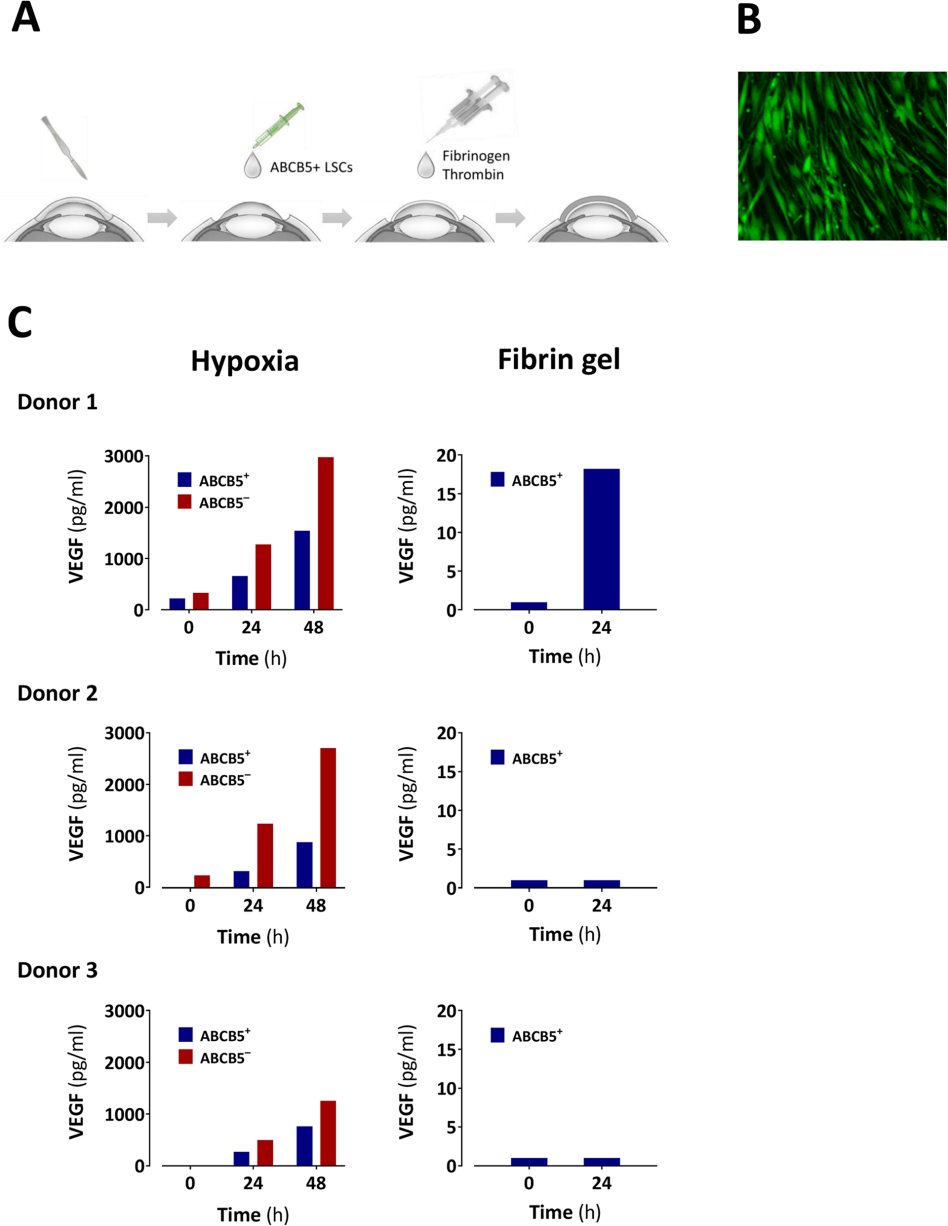
Effect of fibrin gel on ABCB5^+^ limbal stem cells (LSCs). **a** Fibrin gel is used as carrier for transplantation of ABCB5^+^ LSCs onto the debrided cornea. **b** Calcein staining demonstrates viability of ABCB5^+^ LSCs after 72 h of culture in fibrin gel. **c** Fibrin gel does not create hypoxic conditions capable of stimulating VEGF secretion by ABCB5^+^ LSCs in vitro as shown by VEGF secretion after cultivation under hypoxic conditions (as compared to donor-matched ABCB5^−^ limbal cells, left) and in fibrin gel (right). This experiment was performed on LSCs from three donors. Please note different *y*-axes scales between hypoxia and fibrin gel

To rule out potential detrimental effects of the fibrin gel intended as carrier for LSC transplantation, viability and VEGF secretion of ABCB5^+^ LSCs cultured in fibrin gel were investigated. Calcein staining confirmed viability of ABCB5^+^ LSCs after 72 h of culture in fibrin gel (Fig. 4b). Whereas hypoxic culture conditions stimulated ABCB5^+^ LSCs to secrete VEGF into the culture supernatant, VEGF secretion was not stimulated after 24 h culture in fibrin gel (Fig. 4c).

### Biodistribution studies

#### Local biodistribution of ABCB5^+^ LSCs following topical administration after corneal and limbal debridement

Ten (five male, five female) NSG mice with corneal and limbal debridement at the right eye received 5000 ABCB5^+^ LSCs via cell-containing fibrin gel topically onto the debrided cornea/limbus. After 8-week follow-up, mice were necropsied, and local tissues subjected to qPCR analysis for detection of human DNA fragments originating from the applied cells. Positive results are summarized in Table S5 (Additional file 1).

In the application-site tissues, i.e., the right eye’s anterior segment (cornea and lens), quantifiable levels of human cells were recorded in six out of ten samples. Mean cell concentrations were 238 cells/mg tissue (range: 9–876 cells/mg) in male and 66 cells/mg tissue (range: 77–249 cells/mg) in female mice.

In most (57 out of 60) non-target tissue samples, i.e., left eye’s anterior segment, posterior segment and surrounding tissue of both eyes, muzzle with nasal cavities, and naso-lacrimal ducts, no quantifiable levels of human cells were recorded. The positive results recorded in three non-target tissue samples (1 left and 2 right eye’s posterior segment samples) were considered accidental, given the high contamination risk associated with qPCR techniques including the challenging splitting of the eye into anterior and posterior segment. This is supported by the observation that the two quantifiable cell concentrations detected in right eye’s posterior segment samples correlated with the highest detected cell numbers in the corresponding anterior eye segments.

#### Systemic biodistribution of ABCB5^+^ LSCs following subconjunctival injection

Three groups of ten (five male, five female) NSG mice received a subconjunctival injection of 0.5 × 10^6^ ABCB5^+^ LSCs in 25 μl vehicle at the right eye. Mice were followed up for 1, 12, and 20 weeks, respectively, after which they were sacrificed following terminal blood sampling. As control, a fourth group of ten mice was injected with vehicle only and followed up for 20 weeks.

In the animals receiving subconjunctival injections of ABCB5^+^ LSCs, quantifiable levels of human cells were found in 2/30 samples of treated eye and 1/30 samples of treated eye’s surrounding ocular tissue (12 weeks), 1/30 lung samples (1 week), 4/30 skin/subcutis samples (12 weeks, *n* = 1; 20 weeks, *n* = 3), and 1/15 testes samples (1 week) out of 540 tissue samples in total (Table S6 (Additional file 1)). As the cell level detected in the testes sample was very low (initial analysis: 9 cells/mg; eluate re-analysis: below lower limit of quantification (LLOQ); homogenate re-analysis: 5 cells/mg) and no further testes sample at any time point after LSC application tested positive, this result was attributed to contamination during sample processing. In all other tissues tested (see the “Methods” section), no quantifiable levels of human cells were recorded. In the control group, no clearly positive cell level (above LLOQ) was detected in any of the tissues analyzed.

### Toxicity and tumorigenicity studies

#### Single-dose local toxicity of ABCB5^+^ LSCs following topical administration after corneal and limbal debridement

Two groups of ten (five male, five female) NSG mice each with corneal and limbal debridement at the right eye received 5000 ABCB5^+^ LSCs via cell-containing fibrin gel or fibrin gel without cells (control group) topically onto the debrided cornea/limbus. During an 8-week follow-up period, mice were observed regarding mortality, clinical signs, body weight, food consumption, and ophthalmology. Thereafter, mice were sacrificed and subjected to pathological and local histopathological evaluation.

With regard to the investigated parameters, the study did not reveal any adverse effect related to ABCB5^+^ LSCs. One animal in the control group died on day 7 due to anesthesia for tarsorrhaphy suture removal. Ophthalmological findings in the right eye of both control and cell-treated animals included corneal neovascularization and focal or diffuse corneal opacity, which precluded examination of the fundus. Histopathological evaluation of ocular tissue sections revealed minimal or moderate incomplete regenerative changes, sometimes with detachment of the corneal epithelium and fibroblastic changes in the corneal stroma accompanied by stromal degeneration in the right eye. Mucous (goblet) cells and minor subacute inflammatory changes were noted in the corneal epithelium (Table S7 (Additional file 1)). As there were no differences in nature, incidence or severity between LSC-treated and control group, the clinical and histopathological signs observed were attributed to the technical procedures (corneal debridement) and not to the applied cells.

#### Systemic repeated-dose toxicity and tumorigenicity of ABCB5^+^ LSCs following subconjunctival administration

Three groups of NSG mice received either three doses of 0.5 × 10^6^ ABCB5^+^ LSCs in 25 μl HRG (*n* = 20) or 25 μl HRG vehicle without cells (vehicle control, *n* = 20) on days 0, 14, and 28, or 1.25 × 10^4^ HeLa cells in 25 μl BSS on day 0 (positive control, *n* = 10). During a 20-week follow-up period, mice were observed regarding mortality, clinical signs, body weight food consumption, and ophthalmology. Thereafter, animals were sacrificed, and full necropsy and subsequent histopathological evaluation performed.

During the study, vehicle-control mice gained more weight (mean ± SD; males: 5.1 ± 1.5 g, females: 4.1 ± 0.9 g) than LSC-treated mice (males: 4.5 ± 1.4 g, females: 3.0 ± 1.1 g). These differences between groups reached statistical significance only for the female, but not for the male animals. One LSC-treated mouse died after anesthesia for the second application (day 14). Ophthalmologic examinations, performed at days 14, 28, 35, 42, and at week 20, revealed inflammation of the anterior segment of both eyes in one LSC-treated animal on day 14, which had resolved on day 42. Since both, the treated and the untreated eyes were affected, this finding was assumed not related to LSC application. During pathological evaluation, no LSC-related macroscopic and microscopic findings were identified. All macroscopic and microscopic findings in the vehicle- and LSC2 cell-injected mice were considered spontaneous background findings. Therefore, no evidence of any tumor formation was observed. As a positive control to demonstrate our ability to detect subconjunctival tumors, mice received subconjunctival injections of HeLa cells. Two of the HeLa-treated mice died due to anesthesia for ocular examinations on day 28. Six out of the remaining eight animals (75%) developed moderate to extreme tumor masses at the injection site (right eyes) and were prematurely euthanized around week 12. Histopathological evaluation revealed small lung metastases in five of these animals. One animal had also metastases in the brain.

## Discussion

While current LSC-based therapies have resulted in long-term restoration of the corneal epithelium and improvement of visual acuity [11, 14, 18], they are predominantly best suited to treat unilateral disease [42]. In bilateral LSCD, where autologous LSCs are not available for transplantation, allogeneic grafts are required. However, clinical studies using allogeneic limbal tissue transplants have often provided only transient corneal restoration [12, 15, 43], which was suspected to be caused by immunogenic limbal cell subpopulations such as Langerhans cells capable of inducting rejection responses in the recipient. Furthermore, current approaches are often associated with regulatory and logistical obstacles, seeing that the grafts contain variable numbers of LSCs and that the preparations have not been shown to be (cryo-) preservable [42]. We hypothesized that surface marker-based prospective isolation, expansion, and purification of LSCs from deceased donors might overcome these obstacles by precluding the transfer of potentially highly immunogenic cell subpopulations, ensuring defined composition and purity of the cell product, and enabling storage and transportation [4, 42]. In the light of this situation, we strived to develop and validate a GMP-compliant manufacturing process, by which ABCB5^+^ LSCs from cadaveric human limbal tissue can be expanded in vitro, isolated as a homogenous cell population and manufactured as a clinical-grade ATMP (Fig. 1) [44].

Factors that have been suspected to impact on the number or functional characteristics of LSCs isolated from limbal donor tissue are donor age [45] and the duration of tissue preservation until processing [46]. Notara et al. [45] have described age-related changes in limbal stem cell niche topography with a significant reduction in the surface area occupied by LSC niche structures and flattening of the palisades of Vogt occurring after the age of 60 years. However, donor age did not seem to affect the limbal cell yield in our process, since we did not observe a statistically significant correlation between donor age (51–90 years) and cell count or cell adhesion rate in primary cell culture (Figure S3b (Additional file 2)). In line, Sasamoto et al. [47] could not establish a statistically significant association between donor age (24–79 years) and yields of freshly isolated ABCB5^+^ LSCs. Also, we did not find the tissue storage time (normothermic preservation for up to 76 days) to be correlated with cell count or cell adhesion rate in primary cell culture (Figure S3b (Additional file 2)). This has also been described for hypothermic preservation at 4 °C (which is commonly used in the USA and Asia to store corneoscleral discs intended for corneal transplantation [48, 49]), for which no significant association between storage time and the yield of freshly isolated ABCB5^+^ LSCs was reported [47].

During expansion, the cultured cells exhibited normal growth behavior without any signs of overgrowing potential; i.e., once the cells had reached confluence, they slowed down proliferation, which is indicative of physiological contact inhibition (Figure S4 (Additional file 2)). Immunofluorescence characterization of unsegregated cultured limbal cells revealed a marker expression profile that corresponded, for the very most part, to the profile expected for LSCs. Specifically, we detected heterogenous expression of the well-recognized LSC marker p63 [20] and homogenous expression of PAX6, which has been shown to play a critical role in limbal stem cell fate determination [50–52]. Slight expression of the conjunctival epithelium marker CK19 and of vimentin is in line with reports that have detected these proteins on limbal basal cells [17, 53]. Although both proteins have been interpreted as additional limbal stem cell markers [17, 53], we cannot, however, rule out that the vimentin-positive cells in our primary cell cultures instead represent a small proportion of mesenchymal-origin limbal niche cells co-isolated from the limbal tissue due to the use of collagenase instead of dispase digestion [54].

The absence of the filament proteins CK3/12, which are, due to their specific expression in corneal epithelial cells and limbal suprabasal but not basal cells, regarded as markers of corneal epithelial differentiation [17, 53, 55], indicates that the cultivated LSCs have maintained their undifferentiated nature throughout the expansion process. In contrast, the slight positivity for the gap junction protein connexin 43 was unexpected, as this protein was, similar to CK3/12, suggested as a putative negative biomarker of LSCs [56]. As gap junction proteins such as connexin 43 mediate intercellular communication of the human corneal epithelium, the absence of these proteins is considered a prerequisite for LSCs to maintain their stemness, whereas the expression of connexin 43 was suggested to denote differentiation of LSCs into transient amplifying cells [57]. Therefore, it cannot be excluded that a small subpopulation within the cultured limbal cells had undergone early stages of differentiation.

In addition to their role as stem cell markers, the transcription factors p63 and PAX6 served as surrogate parameters to predict the cells’ functional potency [58, 59]. As p63 is critical for LSC maintenance [20] and PAX6 is essential for commitment of LSCs to the corneal epithelial cell lineage [50, 51], these two factors reflect the two endpoints relevant in LSCD therapy; namely, restoration and maintenance of the limbal barrier against invading conjunctival cells and restoration and regular renewal of the corneal epithelium, respectively. In large clinical observations, p63 expression of ex vivo cultured limbal grafts was directly correlated with the therapeutic outcome, with a content of 3% p63^+^ cells appearing as threshold value for clinical transplant success [18]. In our manufacturing process, p63^high^ cells were specifically enriched in the ABCB5^+^ population (Fig. 2b), amounting to a mean p63^high^ cell content of 50% (Fig. 2a). As acceptance criterion for batch release, a p63^+^ cell content of ≥20% was specified (Table 2), which is far above the threshold content for clinical transplant success of 3% p63^+^ cells [18]. Noteworthy, about 83% of the isolated ABCB5^+^ cells that we characterize as “p63^+^” based on positivity for ΔNp63 stain also positive for p63α. Quality control data of GMP-compliantly manufactured cell batches show that 100% of the tested batches exceeded the specifications for p63^+^ and PAX6^+^ cell content (Table 2, Fig. 3).

To ensure safe usage of the final drug product it was crucial to control for potential cellular impurities, most importantly residual melanocytes and Langerhans cells. Melanocytes reside as sporadic cells within the limbus [60], where their proximity to LSCs has suggested a physiological role for stemness maintenance of LSCs [61]. However, melanocytes can transform into melanoma cells, and two recently published single-case reports of donor-transmitted ocular melanoma occurring after keratolimbal allograft transplantation [62, 63] might raise concern of whether our LSC product might confer a melanoma risk to the patient. Furthermore, as ABCB5 was found expressed on melanoma cells [64], one might suspect that, if there indeed were melanoma cells present in the primary culture, sorting for ABCB5^+^ cells could even enrich them during the courses of cell expansion and isolation. Thus, in addition to multiple in-process controls to detect signs of tumorigenic transformation, including control of growth behavior and cell morphology (see Fig. 1 and Table 1), we evaluated the final drug product for expression of the melanocyte antigen MART-1. Whereas positive-control SK-MEL cells stained highly positive for MART-1, no MART-1 could be detected on the cells of the final drug product.

As the limbal region also contains Langerhans cells [17, 65, 66], which have been thought to be associated with graft failure in corneal transplantation [66], we needed to ensure that the final drug product does not contain Langerhans cells either. One may find it surprising that we could not detect any positivity for CD1a already in the corneal rims that served as starting tissue. However, whereas Langerhans cells are consistently observed in fresh limbal tissue [65, 66], rapid depletion of Langerhans cells in corneal and limbal tissue during storage in various storage and culture media has been described, which can lead to total absence already within 1–3 weeks of storage [65–67]. As expected from our Langerhans cell-free starting material, Langerhans cells could also not be detected in the final drug product.

Before being released, each batch of ABCB5^+^ LSCs is subjected to a series of tests to verify conformity with prespecified criteria (Table 2). Except for one batch, which failed the specification for vitality, all batches tested so far fulfilled the specifications defined for batch release (Table 2, Fig. 3), which demonstrates robustness of the entire LSC expansion and isolation process and ensures homogeneity and reliable quality and safety of the released batches.

The next step on the way to clinical in-human usage of in vitro expanded ABCB5^+^ LSCs was to preclinically investigate the engraftment potential and in vivo safety of the cell product. First, we studied the local biodistribution and engraftment behavior of topically administered ABCB5^+^ LSCs to mice having undergone mechanical removal of the entire corneal and limbal epithelia. In the application-site tissues, i.e., the treated eye’s anterior segment (cornea and lens), quantifiable levels of human cells were recorded in six out of ten mice at 8 weeks following cell application (Table S5 (Additional file 1)), indicating that the transplanted cells had indeed engrafted, even in the xenogeneic setting. This is in line with earlier observations using freshly isolated ABCB5^+^ cells from the human limbal tissue in a murine LSCD model [23]. In this study, human-specific β2-microglobulin transcripts were detectable in the corneal tissue of all recipient mice even 13 months after transplantation [23]. Because that study aimed at investigating the cells’ cornea-regenerative potential, the mice had, in contrast to our safety study, received concomitant topical anti-angiogenic therapy using the anti-VEGF antibody bevacizumab. Pre- and post-operative anti-angiogenic therapy has been reported to promote graft survival and to improve therapeutic outcomes in high-risk corneal transplantation and limbal stem cell transplantation [68–70]. Thus, the observation that in our preclinical study engraftment occurred only in six out of ten mice may be attributed to the lack of any concomitant anti-angiogenic therapy rather than to changes of the LSCs’ engraftment potential during in vitro cell expansion.

In contrast to the anterior segment of the treated eye as the target tissue, in virtually none of the non-target tissue samples any quantifiable levels of human cells were recorded (with the 3 out of 60 positive samples being regarded as artifacts, as commented on in the “Results” section). This indicates that no local migration of the transplanted LSCs beyond the anterior eye segment had occurred.

To evaluate the LSCs’ behavior in the worst-case situation, i.e., in the unexpected event that some of the applied cells enter the densely vascularized subconjunctival tissue, we also assessed the systemic biodistribution and persistence potential of our drug product. This study revealed that ABCB5^+^ LSCs that, under worst-case conditions, reach the bloodstream, can occasionally migrate beyond the application-site tissues into the lung and the skin (Table S6 (Additional file 1)). Notably, in the lungs, which are basically considered as first-pass organ for intravenously administered stem cells [71], LSCs were found in one animal after 1 week, but in no animal at later time points. The persistence of LSCs in the skin/subcutis of four animals for up to 20 weeks is difficult to explain, as the skin/subcutis is far different from the physiological LSC niche. However, in total only 8 of 540 (1.5%) tissue samples from cell-treated animals tested positive for human cells above LLOQ. Thus, the study indicates that ABCB5^+^ LSCs would only rarely reside in subconjunctival tissue or migrate to the lung or skin tissue. Notably, this study has addressed a worst-case scenario of subconjunctival cell application, which is neither intended nor expected to occur in the setting of LSC transplantation for LSCD therapy.

Potential toxicity of LCSs was also assessed after both topical administration in the corneal/limbal debridement model and repeated subconjunctival injections. Both studies did not reveal any clinical, pathological, or histopathological signal of cell treatment-related toxicity. A major concern associated with stem cell-based therapies is that the cells may bear a tumorigenic risk, given that stem cells present some characteristics that are also seen in cancer cells, such as long lifespan, self-renewal, and high proliferation rate [72]. In addition, ABCB5 recently was found upregulated in ocular surface squamous neoplasia, although limited sample size and varying ABCB5 expression patterns did not allow proper evaluation of the relationship between ABCB5 expression and tumor formation [73]. Regardless, tumor risk assessment is an essential part in biosafety evaluation, particularly in view of the concomitant immunosuppressive medication that LSC-transplanted LSCD patients will receive. In our preclinical tumorigenicity study, we did not detect any macro- or micropathological signs of tumor formation in NSG mice after repeated subconjunctival injections of ABCB5^+^ LSCs up to 20 weeks. Positive-control mice received a 40-fold lower number of HeLa cells (1.25 × 10^4^ HeLa cells as compared to 0.5 × 10^6^ ABCB5^+^ LSCs). Even with this considerably lower cell dose, 75% of the HeLa-treated animals had developed tumors at the injection site by weeks 7 to 10. In addition, in previously published studies, subconjunctival injection of 0.4 × 10^6^ conjunctival melanoma cells (i.e., a dose similar to that of the ABCB5^+^ LSCs in the present study) to NSG mice resulted in ocular tumor development in 100% of the animals [74, 75]. Together, these findings confirm the ocular tumor susceptibility of the animal model used, which, in turn, counts against a tumor risk of treatment with ABCB5^+^ LSCs. Taken together, the cell dose of 0.5 × 10^6^ LSCs/eye applied in the systemic repeated-dose toxicity/tumorigenicity study was considered as the No observed adverse event level (NOAEL).

Dose calculation for the first-in-human trial of our product was based on the following considerations: Given, according to our product specifications, ≥ 90% viability and ≥ 90% ABCB5^+^ cell content (Table 2), the lowest intended dose of 7.5 × 10^4^ cells/eye will supply at least 565 viable ABCB5^+^ LSCs/mm^2^ corneal surface (assuming a mean human corneal diameter of 11.7 mm [76], which corresponds to 107.5 mm^2^ corneal surface area). This cell dose is more than 15-fold the amount of 36 viable ABCB5^+^ LSCs/mm^2^ corneal surface, corresponding to the cell dose of 255 viable ABCB5^+^ LSCs per mouse eye that was previously shown capable of restoring the cornea upon grafting to LSC-deficient mice [23] (assuming a mean mouse corneal diameter of 3 mm [77], which corresponds to 7.1 mm^2^ corneal surface). On the other end, the highest intended dose of 1.2 × 10^6^ cells per eye (corresponding to 1.1 × 10^4^ cells/mm^2^) is 6.4-fold lower than the NOAEL of 0.5 × 10^6^ cells per mouse eye, which corresponds to 7 × 10^4^ cells/mm^2^.

Finally, for therapeutic efficacy, it will be crucial that the cells’ viability and biological activity are not detrimentally impacted by the fibrin gel used as carrier for cell transplantation onto the debrided cornea. Calcein staining of ABCB5^+^ LSCs after culture for 72 h in fibrin gel confirmed that the viability of the cells is preserved in the fibrin carrier (Fig. 4b). Regarding the cells’ biological activity, it had to be ruled out that the fibrin gel could create a hypoxic environment that, in turn, would stimulate the LSCs to secrete proangiogenic factors such as VEGF, as is known, e.g., for ABCB5^+^ mesenchymal stem cells [31]. Such adaptive secretion of proangiogenic factors would facilitate angiogenesis and thus jeopardize therapeutic success. However, the fibrin gel did not seem to induce hypoxic conditions, as VEGF secretion by ABCB5^+^ LSCs was not stimulated after 24 h culture in fibrin gel (Fig. 4c).

## Conclusions

Taken together, we demonstrate herein that human ABCB5^+^ LSCs, derived from cadaveric corneal tissue, can be reliably expanded, prospectively enriched, and manufactured as a GMP-conform ATMP that contains comparably high percentages of cells expressing transcription factors critical for LSC stemness maintenance (p63 [20]) and corneal epithelial differentiation (PAX6 [50, 51]). In addition, our preclinical study program has revealed a favorable biodistribution and safety profile of the final product in view of its intended clinical use. Building upon these data in conjunction with the previously shown cornea-restoring capacity of human ABCB5^+^ LSCs in animal models of LSCD [22, 28], we provide an advanced allogeneic LSC-based treatment strategy that shows promise for replenishment of the patient’s LSC pool, recreation of a functional barrier against invading conjunctival cells and restoration of a transparent, avascular cornea. Recently, the ATMP has received Orphan Drug designation from the U.S. Food and Drug Administration (FDA) [78] and the European Medicines Agency (EMA) [79] and was included in the FDA Fast Track program. At present, it is being tested in an international, multicentric clinical trial.

## Supplementary Information

**Additional file 1: Table S1.** Tests and specifications for drug substance batch and final drug product release. **Table S2.** Antibodies used for immunofluorescence evaluation. **Table S3.** Primers and probes. **Table S4.** Main inclusion and exclusion criteria of the clinical trial. **Table S5.** Positive qPCR results from the local biodistribution study. **Table S6.** Positive qPCR results from the systemic biodistribution study. **Table S7.** Histopathological findings from the local toxicity study.

**Additional file 2: Figure S1.** Representative morphological images of a primary cell culture at early and late passage. **Figure S2.** Determination of ABCB5^+^ cell content. **Figure S3.** Expansion process validation. **Figure S4.** Growth behavior of ABCB5^+^ limbal stem cells during culture. **Figure S5.** Immunohistochemical characterization of the unsegregated limbal cell culture. **Figure S6.** Immunohistochemical staining for ΔNp63 of human corneal rim cryosections and of bead-isolated ABCB5^+^ cells. **Figure S7.** Expression of the ΔNp63α isoform of the p63 transcription factor by ABCB5^+^ LSCs as evidenced by co-staining for N-terminally truncated p63 (ΔNp63) and p63 alpha isoforms (p63α).

## Data Availability

The datasets generated and/or analyzed during the current study are available from the corresponding author on reasonable request. For requests regarding ABCB5 mAb availability contact Markus H. Frank, Markus.Frank@childrens.harvard.edu.

## Abbreviations

ABCB5: ATP-Binding Cassette Transporter, Subfamily B, Member 5
BSS: Balanced salt solution
CFSE: Carboxyfluorescein diacetate succinimidyl ester
CK: Cytokeratin
DAPI: 4′,6-Diamidino-2-phenylindole
GMP: Good Manufacturing Practice
HRG: Ringer’s lactate solution containing 2.5% human serum albumin and 0.4% glucose
LLOQ: Lower limit of quantification
LSC: Limbal stem cell
LSCD: Limbal stem cell deficiency
MART-1: Melanoma antigen recognized by T cells 1
PFA: Paraformaldehyde
PAX6: Paired box protein 6
p63: Tumor protein 63
PBS: Phosphate-buffered saline
VEGF: Vascular endothelial growth factor
