# Supplementary material for: Process development and safety evaluation of ABCB5+ limbal stem cells as advanced-therapy medicinal product to treat limbal stem cell deficiency
Source: Stem Cell Res Ther. 2021 Mar 19;12:194. doi: 10.1186/s13287-021-02272-2 (PMC7980611; doi:10.1186/s13287-021-02272-2)
Supplement: Supplementary file 1 — Additional file 1: Table S1. Tests and specifications for drug substance batch and final drug product release. Table S2. Antibodies used for immunofluorescence evaluation. Table S3. Primers and probes. Table S4. Main inclusion and exclusion criteria of the clinical trial. Table S5. Positive qPCR results from the local biodistribution study. Table S6. Positive qPCR results from the systemic biodistribution study. Table S7. Histopathological findings from the local toxicity study. [file 13287_2021_2272_MOESM1_ESM.pdf]

Table S1 Tests and specifications for drug substance batch and final drug product release

Parameter	Method	Specification	
		**Drug substance** (isolated ABCB5$^+$ LSCs)	**Final drug product** (ABCB5$^+$ LSCs in HRG, 2.5×10^5, 1×10^6, 2.67×10^6 or 4×10^6 cells/ml, depending on the cell dose to be administered)
ABCB5$^+$ cell content	Flow cytometry	≥ 90%	≥ 90%[a)]
Mycoplasma	Nucleic acid amplification test (2.6.7/2.6.21 Ph. Eur.)	not detectable (< 10 CFU/ml)	not detectable (< 10 CFU/ml)[a)]
Endotoxin level	Limulus amebocyte lysate test (2.6.14 Ph. Eur.)	≤ 2 EU/ml	≤ 2 EU/ml[a)]
Cell vitality	Flow cytometry (2.7.29/2.7.24 Ph. Eur.)	≥ 90%	≥ 75%
Cell viability	Flow cytometry (2.7.29 Ph. Eur.)	≥ 90%	≥ 90%[a)]
Bead residues	Flow cytometry	≤ 0.5%	≤ 0.5%[a)]
Microbiological control	BacT/ALERT® (adapted to 2.6.27 Ph. Eur.)	no growth	no growth
p63$^+$ cell content	Immunofluorescence	≥ 20%	≥ 20%[a)]
PAX6$^+$ cell content	Immunofluorescence	≥ 50%	≥ 50%[a)]

HRG Ringer's lactate solution containing human serum albumin and glucose, *LSCs* limbal stem cells, *Ph. Eur.* European Pharmacopoeia

[a]Parameter is not tested in the final drug product; value is adopted from drug substance release testing. Transferability of the result from drug substance release testing onto the final drug product was demonstrated in stability studies and method validations.

Table S2 Antibodies used for immunofluorescence evaluation

Antibody	Host species	Dilution	Supplier (Catalogue number)
**Primary antibodies**			
anti-ΔNp63	goat	1:40	R&D Systems/Bio-Techne (AF1916)
anti-p63α	rabbit	1:30	Cell Signaling (13109S)
anti-CK3/12	mouse	1:100	Abcam (ab68260)
anti-CK19	rabbit	1:100	Abcam (ab52625)
anti-PAX6	rabbit	1:33	Sigma-Aldrich/Merck (HPA030775)
anti-Vimentin	goat	1:100	Santa Cruz Biotechnology (sc-7557)
anti-Connexin 43	rabbit	1:100	Abcam (ab11370)
anti-MART-1	mouse	1:40	Abcam (ab785)
PE/Dazzle™ 594-anti-CD1a	mouse	1:50	Biolegend (300131) (Clone HI149)
Alexa Fluor® 647-anti-CD1a	mouse	1:50	Biolegend (300116) (Clone HI149)
**Secondary antibodies**			
Alexa Fluor® 594-anti-rabbit IgG	donkey	1:500	Biolegend (406418)
Alexa Fluor® 488-anti-mouse IgG (for CK3/12)	donkey	1:500	Molecular Probes™/Thermo Fisher (A-21202)
Alexa Fluor® 488-anti-goat IgG	donkey	1:500	Molecular Probes™/Thermo Fisher (A-11055)
Alexa Fluor® 594-anti-mouse IgG (for MART-1)	goat	1:500	Biolegend (405326)

Table S3 Primers and probes

**Reagent**	**Sequence (5'→3')**
**Human-specific**	
Forward primer ACC-25	GGGATAATTTCAGCTGACTAAACAG
Reverse primer ACC-26	AAACGTCCACTTGCAGATTCTA
Probe ACC-27	FAM-CACGTTTGAAACACTCTTTTTGCA-BHQ-1
**Mouse-specific**	
Forward primer ACC-28	TACCTGCAGCTGTACGCCAC
Reverse primer ACC-29	GCCAGGAGAATGAGGTGGTC
Probe ACC-30	TAMRA-CCTGCTGCTTATCGTGGCTG-BHQ-2

All primers and probes were from Microsynth (Balgach, Switzerland)

Table S4 Main inclusion and exclusion criteria of the clinical trial

Main inclusion criteria

- Age 18–85 years
- Secondary LSCD (causative insult had occurred at least 6 months prior to inclusion)
- Corneal vascularization of at least two quadrants, involving central cornea

Main exclusion criteria

- Compromised eyelid mobility and/or symblepharon
- Eyelid malposition
- Active ocular, intraocular, periocular or systemic infection/inflammation
- Tumor disease or history of tumor disease
- Active ocular neoplastic disease
- Corneal erosion or ulcer > 4 mm^2
- History of glaucoma
- Contraindications to trial-related procedures/agents
- Intraocular pressure ≥ 30 mm Hg
- History or clinical signs of stroke or transient ischemic attacks

Table S5 Positive qPCR results from the local biodistribution study[a)]

**Tissue**		**Sex**	**Cell concentration (human cells/mg tissue)**
Target tissue	Anterior segment (cornea and lens) of the treated (right) eye	Male	55
		Male	876
		Male	9
		Male	247
		Female	77
		Female	249
Non-target tissues[b)]	Posterior segment (retina, sclera and optic nerve) of the treated (right) eye	Male	250
		Male	48
	Posterior segment (retina, sclera and optic nerve) of the untreated left eye	Female	22

[a]Shown are all positive tissue samples out of 10 target tissue samples (anterior segment of the treated eye) and 60 non-target tissue samples (anterior segment of the untreated eye; posterior segment of the treated and of the untreated eye, respectively; surrounding tissue of the treated and of the untreated eye, respectively; and muzzle with nasal cavities/nasolacrimal ducts) in total from 10 mice.

[b]The three positive results in non-target tissues were from animals that had also positive results in the target tissue.

Table S6 Positive qPCR results from the systemic biodistribution study[a)]

Tissue	Sex	Time of sacrifice (weeks post-LSC treatment)	Analysis	Cell concentration (human cells/ mg tissue)[b)]	LLOQ (human cells/ mg tissue)
Right (treated) eye	M	12	Initial analysis<br>DNA eluate re-analysis<br>Homogenate re-analysis	detected<br>detected<br>8	8
	F	12	Initial analysis<br>DNA eluate re-analysis<br>Homogenate re-analysis	detected<br>detected<br>8	
Surrounding ocular tissue of the right (treated) eye	F	12	Initial analysis<br>DNA eluate re-analysis<br>Homogenate re-analysis	13<br>47<br>44	8
Lung	M	1	Initial analysis<br>DNA eluate re-analysis<br>Homogenate re-analysis	6<br>8<br>detected	5
Skin/subcutis	M	12	Initial analysis<br>DNA eluate re-analysis<br>Homogenate re-analysis	5<br>14<br>8	5
	M	20	Initial analysis<br>DNA eluate re-analysis<br>Homogenate re-analysis	42<br>40<br>25	
	F	20	Initial analysis<br>DNA eluate re-analysis<br>Homogenate re-analysis	10<br>8<br>5	
	F	20	Initial analysis<br>DNA eluate re-analysis<br>Homogenate re-analysis	5<br>7<br>11	
Testes	M	1	Initial analysis<br>DNA eluate re-analysis<br>Homogenate re-analysis	9<br>detected<br>5	5

LLOQ Lower limit of quantification, *M* Male, *F* Female

[a]Shown are all positive tissue samples out of 540 tissue samples in total from 30 mice

[b]Signals below LLOQ are designated as “detected”

Table S7 Histopathological findings from the local toxicity study

**Finding (right eye)**<br>Grade	**Number of affected animals**	
	**Control (fibrin carrier only) n=9[a)]**	**ABCB5^{+} LSCs (5000 per animal) n=10**
**Detachment of corneal epithelium**		
Minimal	1	2
Slight	5	2
Moderate	1	0
**Corneal epithelium thinning**		
Minimal	5	3
Slight	4	7
**Corneal stromal degeneration**		
Minimal	0	1
Slight	4	4
Moderate	0	1
**Multinucleate cells, corneal stroma**		
Minimal	1	2
**Fibroplasia/neovascularization, corneal stroma**		
Minimal	5	8
Slight	3	1
Moderate	0	1
**Mucous cells in corneal epithelium**		
Minimal	6	8
**Corneal stromal edema, diffuse**		
Minimal	1	0
Moderate	0	1
**Inflammatory cell infiltration, corneal stroma**		
Minimal	2	5
**Single cell necrosis**		
Minimal	6	4

[a]One animal in the control group died prematurely (day 7) due to anesthesia for removal of the tarsorrhaphy suture
