# Supplementary material for: Process development and safety evaluation of ABCB5+ limbal stem cells as advanced-therapy medicinal product to treat limbal stem cell deficiency
Source: Stem Cell Res Ther. 2021 Mar 19;12:194. doi: 10.1186/s13287-021-02272-2 (PMC7980611; doi:10.1186/s13287-021-02272-2)
Supplement: Supplementary file 2 — Additional file 2: Figure S1. Representative morphological images of a primary cell culture at early and late passage. Figure S2. Determination of ABCB5+ cell content. Figure S3. Expansion process validation. Figure S4. Growth behavior of ABCB5+ limbal stem cells during culture. Figure S5. Immunohistochemical characterization of the unsegregated limbal cell culture. Figure S6. Immunohistochemical staining for ΔNp63 of human corneal rim cryosections and of bead-isolated ABCB5+ cells. Figure S7. Expression of the ΔNp63α isoform of the p63 transcription factor by ABCB5+ LSCs as evidenced by co-staining for N-terminally truncated p63 (ΔNp63) and p63 alpha isoforms (p63α). [file 13287_2021_2272_MOESM2_ESM.pptx]

## Slide 1
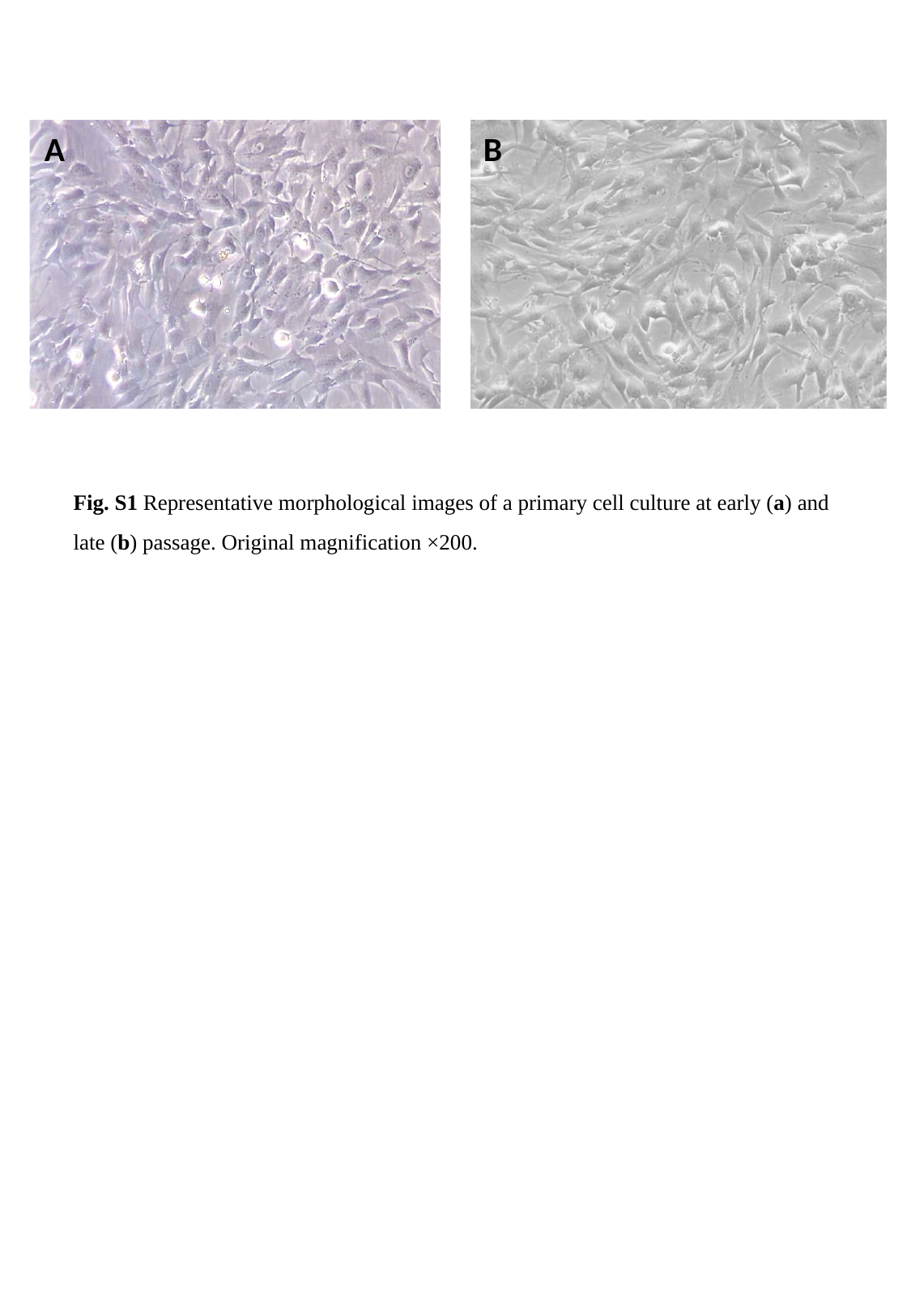

A
B
Fig. S1 Representative morphological images of a primary cell culture at early (a) and late (b) passage. Original magnification ×200.

## Slide 2
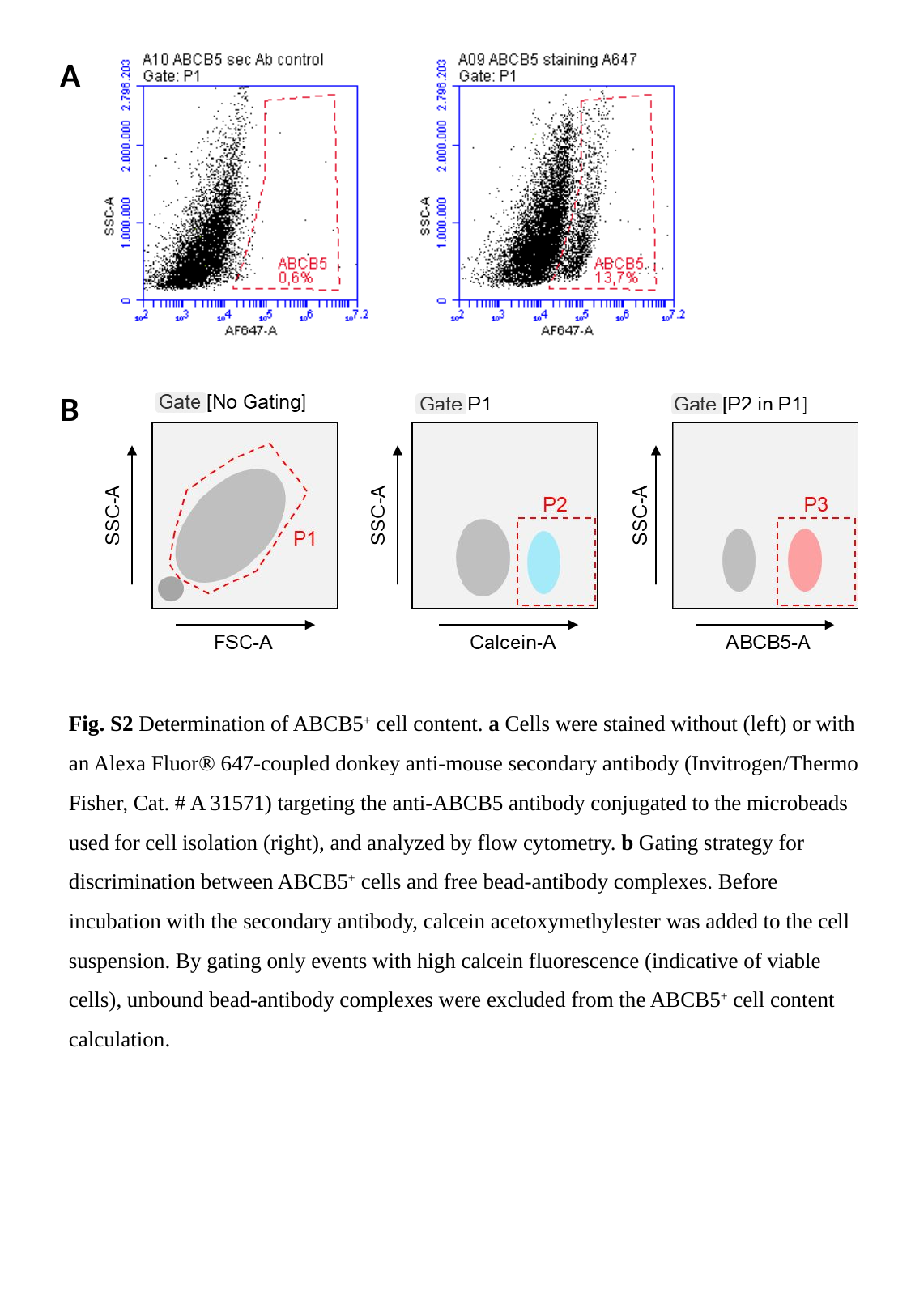

A
B
Fig. S2 Determination of ABCB5+ cell content. a Cells were stained without (left) or with an Alexa Fluor® 647-coupled donkey anti-mouse secondary antibody (Invitrogen/Thermo Fisher, Cat. # A 31571) targeting the anti-ABCB5 antibody conjugated to the microbeads used for cell isolation (right), and analyzed by flow cytometry. b Gating strategy for discrimination between ABCB5+ cells and free bead-antibody complexes. Before incubation with the secondary antibody, calcein acetoxymethylester was added to the cell suspension. By gating only events with high calcein fluorescence (indicative of viable cells), unbound bead-antibody complexes were excluded from the ABCB5+ cell content calculation.

## Slide 3
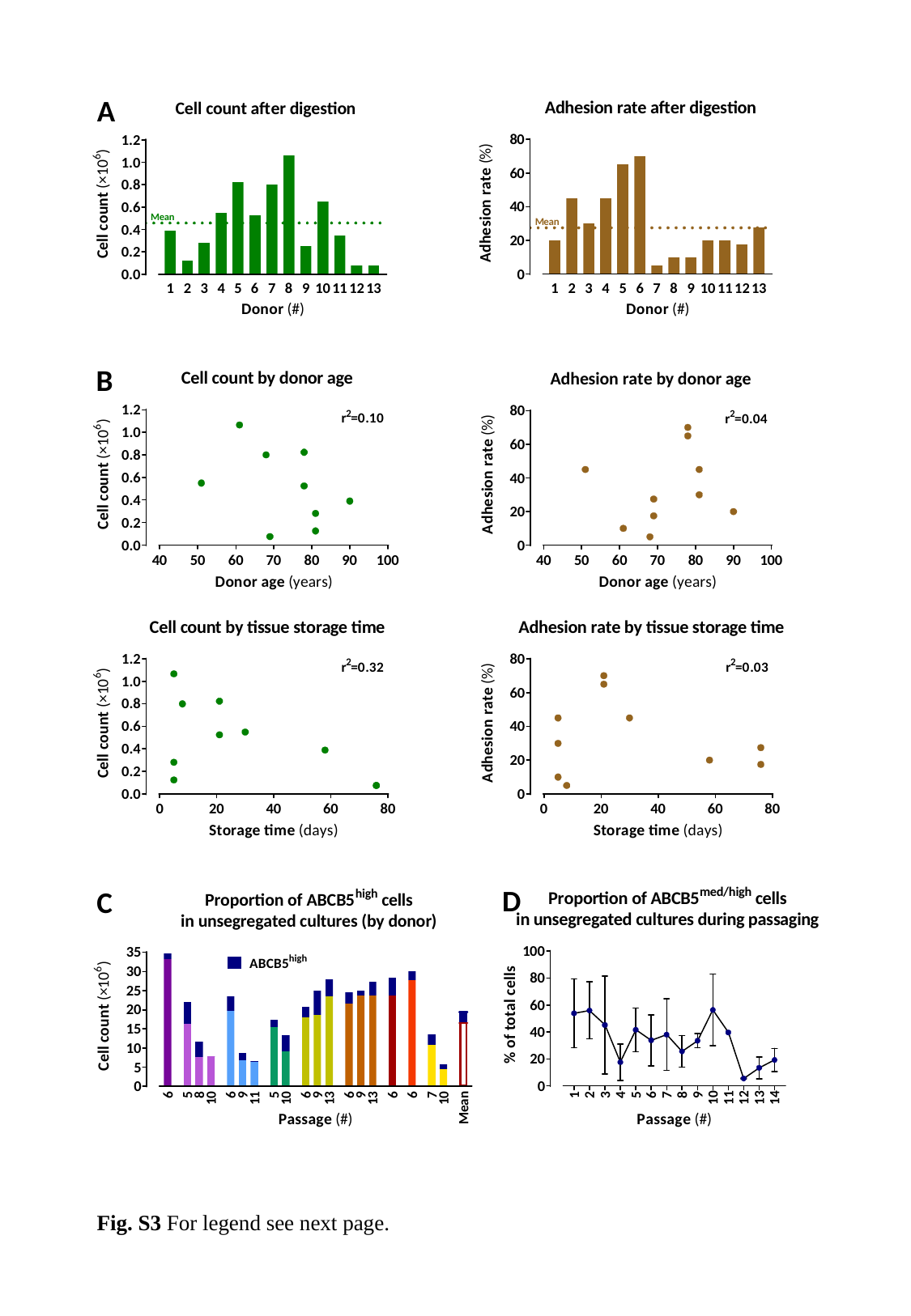

Fig. S3 For legend see next page.

## Slide 4
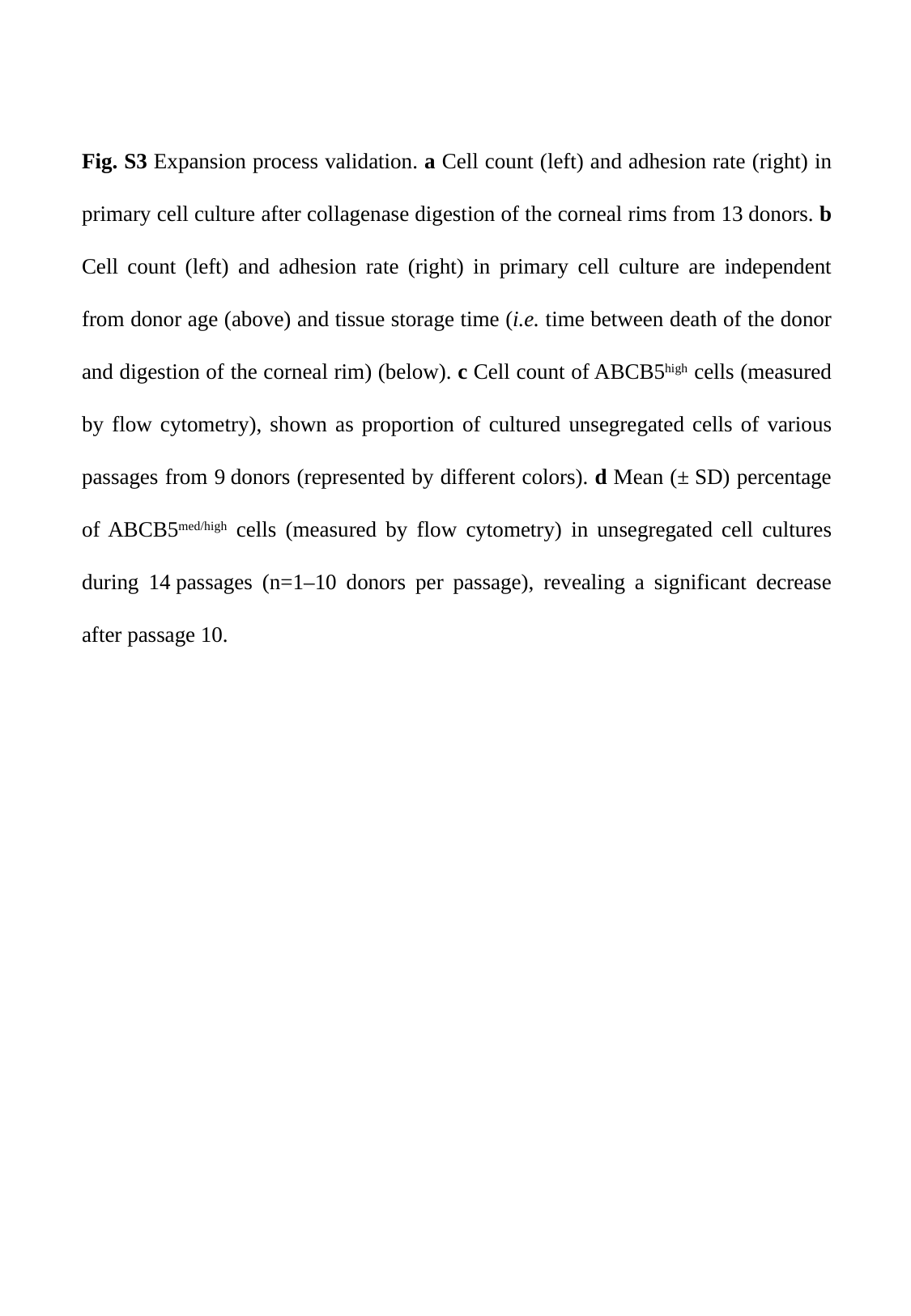

Fig. S3 Expansion process validation. a Cell count (left) and adhesion rate (right) in primary cell culture after collagenase digestion of the corneal rims from 13 donors. b Cell count (left) and adhesion rate (right) in primary cell culture are independent from donor age (above) and tissue storage time (i.e. time between death of the donor and digestion of the corneal rim) (below). c Cell count of ABCB5high cells (measured by flow cytometry), shown as proportion of cultured unsegregated cells of various passages from 9 donors (represented by different colors). d Mean (± SD) percentage of ABCB5med/high cells (measured by flow cytometry) in unsegregated cell cultures during 14 passages (n=1–10 donors per passage), revealing a significant decrease after passage 10.

## Slide 5
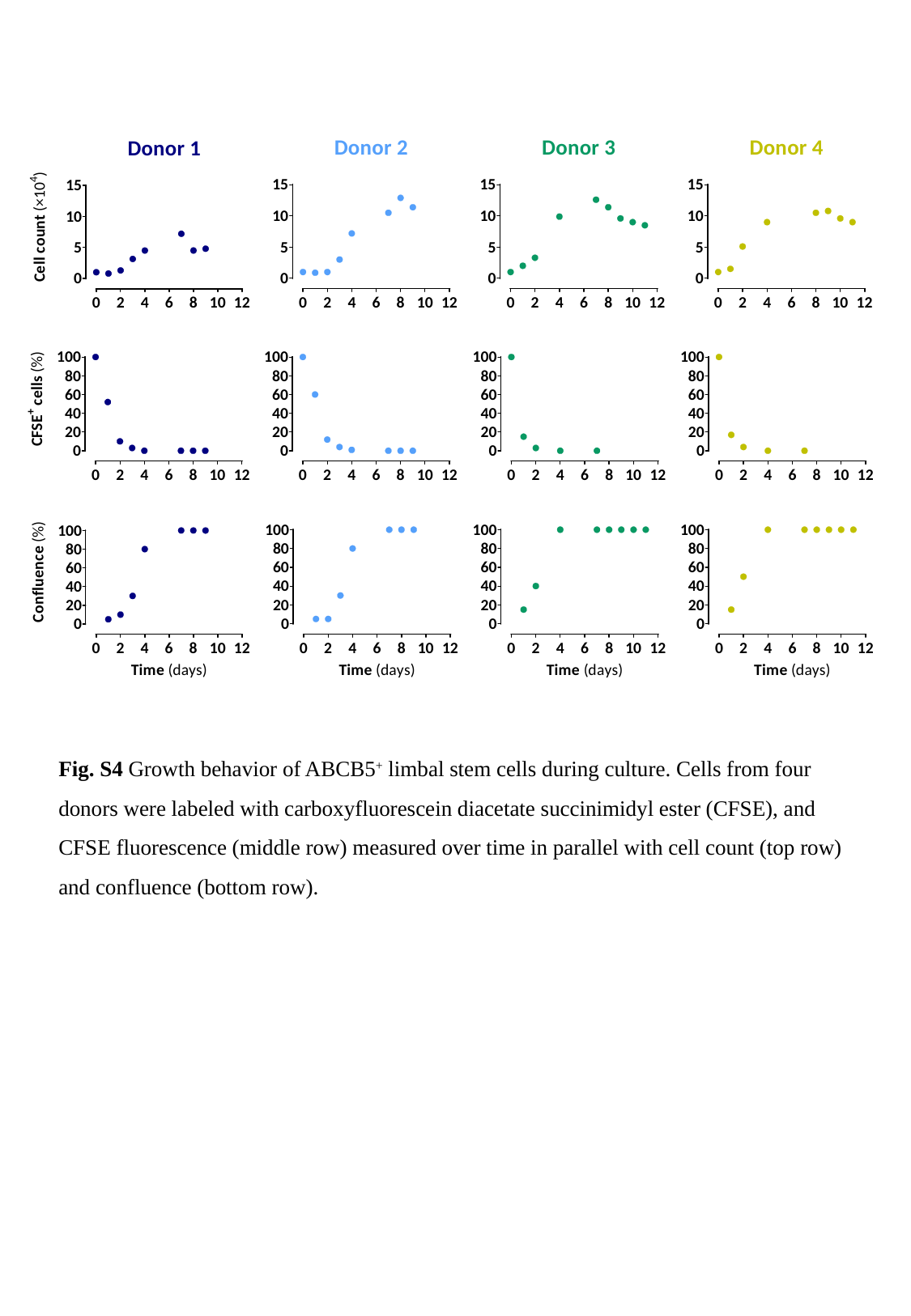

Fig. S4 Growth behavior of ABCB5+ limbal stem cells during culture. Cells from four donors were labeled with carboxyfluorescein diacetate succinimidyl ester (CFSE), and CFSE fluorescence (middle row) measured over time in parallel with cell count (top row) and confluence (bottom row).

## Slide 6
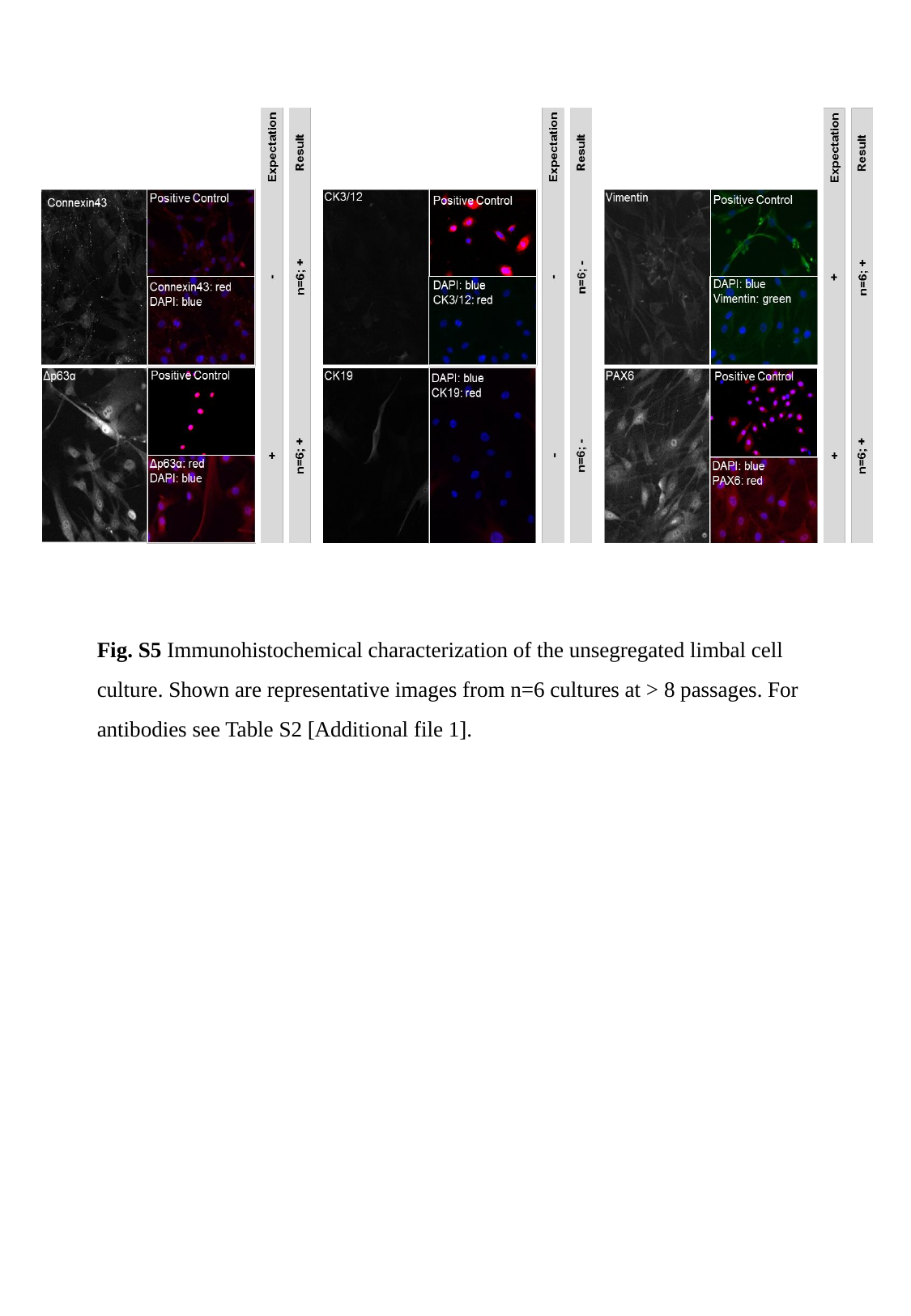

Fig. S5 Immunohistochemical characterization of the unsegregated limbal cell culture. Shown are representative images from n=6 cultures at > 8 passages. For antibodies see Table S2 [Additional file 1].

## Slide 7
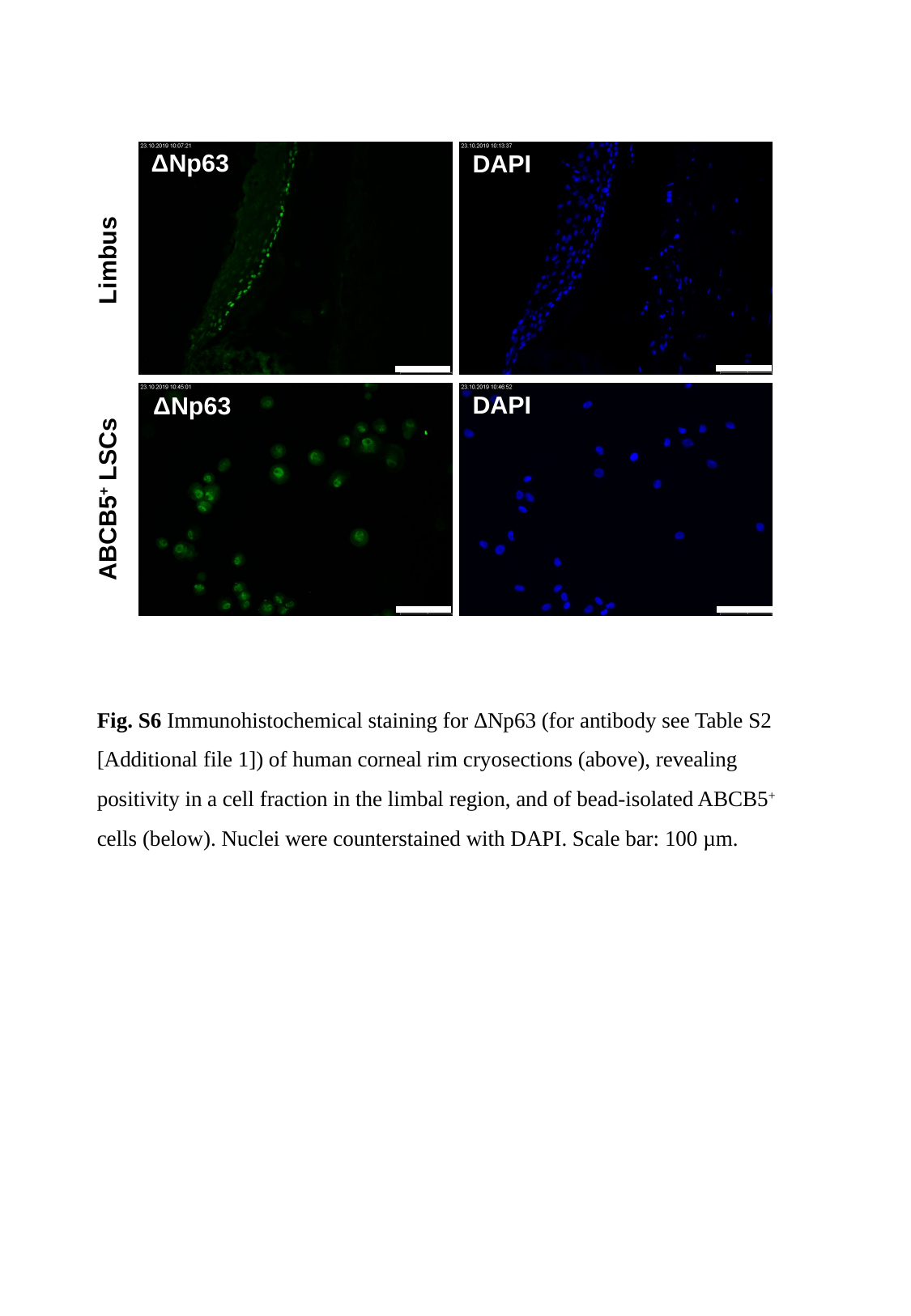

ΔNp63
DAPI
Limbus
DAPI
ΔNp63
ABCB5+ LSCs
Fig. S6 Immunohistochemical staining for ΔNp63 (for antibody see Table S2 [Additional file 1]) of human corneal rim cryosections (above), revealing positivity in a cell fraction in the limbal region, and of bead-isolated ABCB5+ cells (below). Nuclei were counterstained with DAPI. Scale bar: 100 µm.

## Slide 8
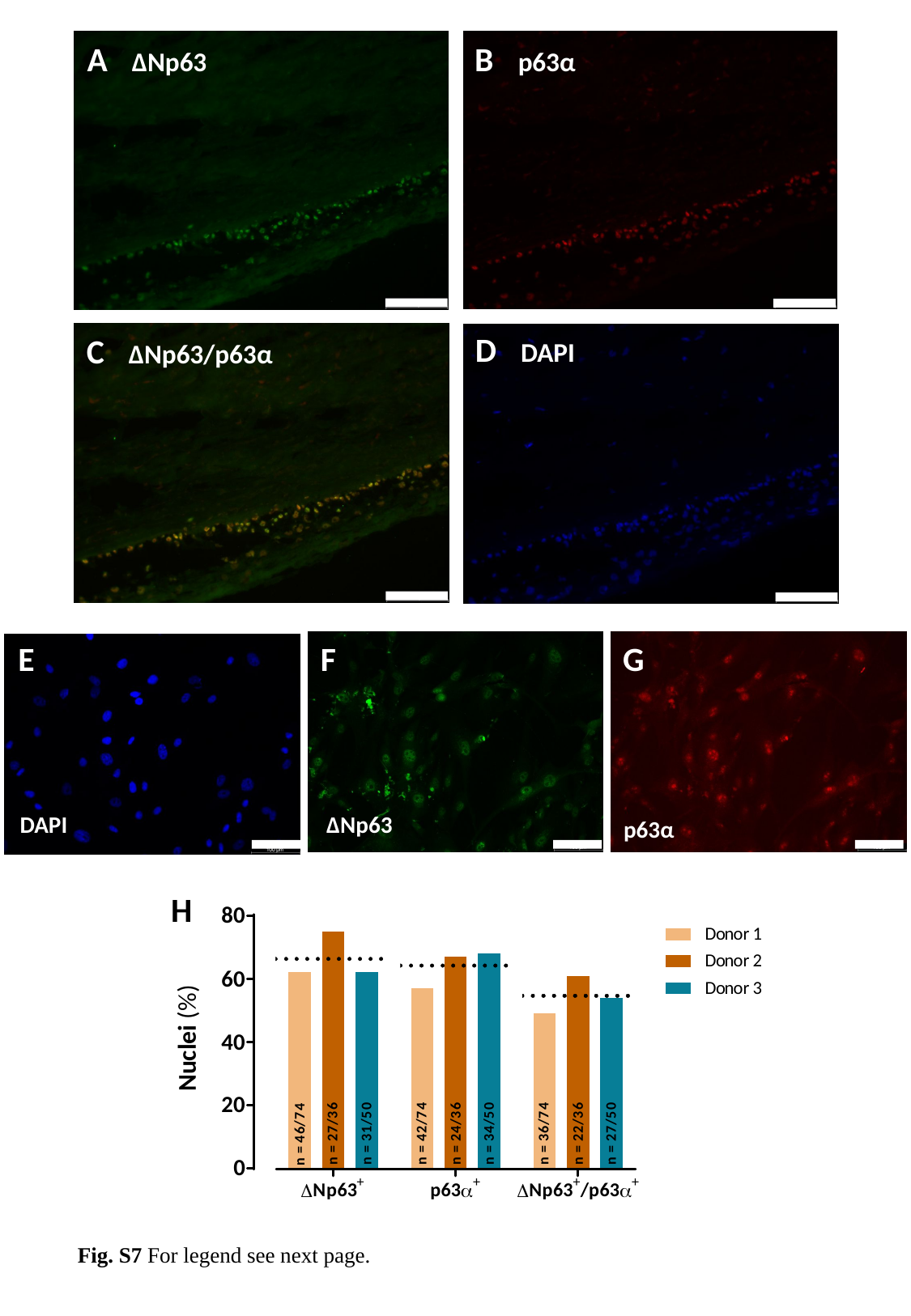

B p63α
A ΔNp63
D DAPI
C ΔNp63/p63α
E
F
G
DAPI
ΔNp63
p63α
H
Fig. S7 For legend see next page.

## Slide 9
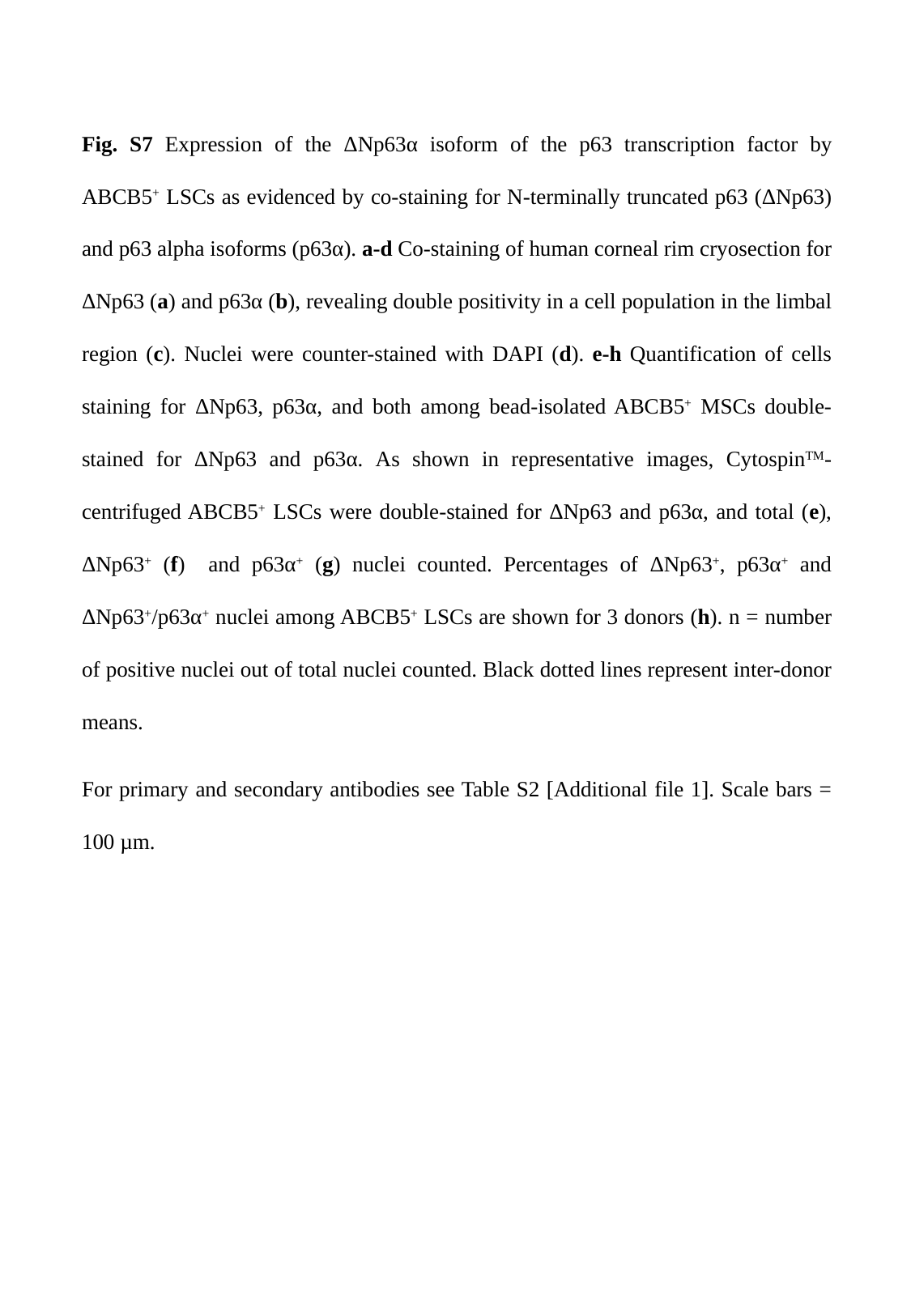

Fig. S7 Expression of the ΔNp63α isoform of the p63 transcription factor by ABCB5+ LSCs as evidenced by co-staining for N-terminally truncated p63 (ΔNp63) and p63 alpha isoforms (p63α). a-d Co-staining of human corneal rim cryosection for ΔNp63 (a) and p63α (b), revealing double positivity in a cell population in the limbal region (c). Nuclei were counter-stained with DAPI (d). e-h Quantification of cells staining for ΔNp63, p63α, and both among bead-isolated ABCB5+ MSCs double-stained for ΔNp63 and p63α. As shown in representative images, CytospinTM-centrifuged ABCB5+ LSCs were double-stained for ΔNp63 and p63α, and total (e), ΔNp63+ (f) and p63α+ (g) nuclei counted. Percentages of ΔNp63+, p63α+ and ΔNp63+/p63α+ nuclei among ABCB5+ LSCs are shown for 3 donors (h). n = number of positive nuclei out of total nuclei counted. Black dotted lines represent inter-donor means.
For primary and secondary antibodies see Table S2 [Additional file 1]. Scale bars = 100 µm.
